# Innovative Metasurface Colorimetric Cell‐on‐a‐Chip Sensor for Continuous, Label‐Free, Non‐Destructive Assessment of Intestinal Barrier Dynamics

**DOI:** 10.1002/advs.202523118

**Published:** 2026-01-28

**Authors:** Youqian Chen, Wen Li, Jiaying Feng, Yue Shu, Yihui Yang, Yuyan Wang, Rui Li, Hanlin Zhou, Xinfan Yang, Yuxue Guo, Mingqian Chen, Wenjun Hu, Gang Logan Liu, Liping Huang, Yanan Li

**Affiliations:** ^1^ School of Food Science and Pharmaceutical Engineering State Key Laboratory of Microbial Technology Nanjing Normal University Nanjing China; ^2^ College of Life Science and Technology Huazhong University of Science and Technology Wuhan China; ^3^ School of Biomedical Engineering Tsinghua University Beijing China; ^4^ College of Life Science and Technology Mudanjiang Normal University Mudanjiang China

**Keywords:** affinity detection, cell‐on‐a‐chip, intestinal cell barrier, live‐cell imaging, MetaSPR biosensor, real‐time cell monitoring

## Abstract

Assessing intestinal barrier function in organ‐ and cell‐on‐a‐chip models is crucial for studying intestinal diseases and advancing drug development. However, current technologies lack methods for continuous, non‐destructive monitoring of intestinal barrier dynamics. In this study, we developed a real‐time, label‐free, high‐throughput cell‐on‐a‐chip system based on a metasurface plasmon resonance colorimetric sensor (MetaSPRCS) to evaluate intestinal barrier function. Integrating MetaSPRCS with transmission microscopy enabled real‐time visualization of cell adhesion, proliferation, and intestinal barrier layer formation, disruption, and repair. The MetaSPRCS platform non‐destructively detects subtle epithelial cell damage caused by low ethanol concentrations, with a two‐fold higher sensitivity than that of CCK‐8 assays. In an alcohol‐induced intestinal barrier injury model, MetaSPRCS validated a reliable evaluation of Dihydroquercetin (DHQ), consistent with endpoint methods. Animal experiments confirmed its ability to predict in vitro‐to‐in vivo dose conversion of DHQ, offering promising guidance for the administration of novel cell‐repairing food ingredients. This study provides a high‐throughput, visual, non‐destructive tool for assessing intestinal homeostasis, establishing a preliminary dose conversion relationship between in vitro evaluation experiments and in vivo animal administration.

## Graphical abstract

We developed a real‐time, label‐free, high‐throughput cell‐on‐a‐chip system based on a metasurface plasmon resonance colorimetric sensor (MetaSPRCS) to evaluate intestinal barrier function. This platform enables continuous monitoring of cell adhesion, proliferation, and barrier layer formation, disruption, and repair. This study provides a novel tool for assessing intestinal homeostasis and guiding the application of cell‐repairing food ingredients.

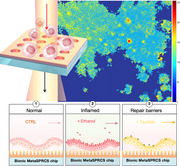

## 1 Introduction

The intestinal barrier is a critical physiological barrier in the human body, effectively preventing the invasion of pathogens and harmful substances while facilitating nutrient absorption [1]. Its integrity is essential for intestinal homeostasis, immune regulation, and protection against diseases such as inflammatory bowel disease, making the barrier a major focus of biomedical research [2]. Currently, common methods for assessing intestinal barrier function include histological staining, (3‐(4,5‐dimethylthiazol‐2‐yl)‐2,5‐diphenyltetrazolium bromide (MTT) assays, crystal violet staining, the lactulose/mannitol test, and serum endotoxin detection [3]. Although these methods provide valuable insights into the intestinal barrier function to a certain extent, they have notable limitations. For instance, MTT assays are end‐point measurements that do not support dynamic monitoring [4, 5]. The lactulose/mannitol test and serum endotoxin detection can provide quantitative information; however, they exhibit limited sensitivity to low‐concentration injury factors and do not offer real‐time monitoring [6, 7]. Moreover, most of these traditional methods are cytotoxic to some extent, which can compromise the normal physiological state of cells. Therefore, there is an urgent need to develop label‐free, highly sensitive methods capable of real‐time monitoring of dynamic changes in the intestinal barrier.

The dynamic integrity of the intestinal barrier is crucial for nutrient absorption, immune homeostasis, and oral bioavailability of drugs. Subtle and rapid alterations in barrier function often precede the onset of inflammatory bowel disease, metabolic syndrome, and alcohol‐induced damage. However, existing methods such as trans‐epithelial resistance, sugar probe permeability, or endpoint colorimetric assays cannot avoid the phototoxicity and bleaching associated with fluorescent labeling, nor can they continuously capture early, reversible barrier disturbances at the live‐cell level. This lack of reliable data for in vitro‐to‐in vivo dose conversion has become a significant bottleneck in precision nutrition and drug development [8]. This technological gap highlights the urgent need for label‐free platforms that can facilitate non‐disruptive monitoring of cellular dynamics over the entire cell life cycle.

Surface plasmon resonance (SPR) is a well‐established technique for real‐time monitoring of molecular interactions and biological processes [9]. SPR technology has recently gained widespread use in the biomedical field owing to its high sensitivity, label‐free detection, and real‐time monitoring capabilities [10, 11, 12, 13]. However, traditional SPR technology is constrained by the need for complex optical equipment, high costs, and limited suitability for long‐term dynamic monitoring. Additionally, their insufficient sensitivity and spatial resolution further impede their application in the study of complex biological systems, such as the intestinal cell barrier. In contrast, metasurface plasmon resonance (MetaSPR) technology, characterized by its distinct nanostructured design and superior optical properties, has demonstrated high potential for various applications [14, 15]. By engineering nanostructured arrays, the MetaSPR chip significantly enhances light‐biomolecule interaction, improving detection sensitivity. This innovative design eliminates the need for traditional complex prism devices, fostering the miniaturization of MetaSPR‐based detection equipment technology and facilitating its easy placement in a cell culture incubator, streamlining relevant experiments and research efforts [16, 17]. The MetaSPR technology has been successfully applied in numerous crucial fields [18, 19, 20, 21, 22], including nucleic acid detection [23], affinity analysis [24], and food safety testing [25]. These optical biosensors are rapidly progressing towards portability and commercial applications owing to their high sensitivity, throughput, time efficiency, and cost effectiveness [26, 27, 28]. However, current MetaSPR applications primarily focus on molecular detection, with limited adaptation for comprehensive cell behavior analysis [29, 30]. Recent studies have highlighted the potential of nanoplasmonic biosensors for highly sensitive and specific detection of biological molecules. For example, Biswas et al. developed a nanoplasmonic aptasensor for detecting dopamine with high sensitivity in complex biological samples [31]. Ansaryan et al. introduced a multimodal nanoplasmonic and fluorescence imaging system that enables simultaneous monitoring of intracellular and extracellular dynamics at the single‐cell level [32]. Zhu et al. proposed a 3D nanoplasmonic biosensor for detecting filopodia in cells, demonstrating its potential for studying cellular morphological changes [33]. These works collectively underscore the power of nanoplasmonic technologies in advancing our understanding of cellular and molecular processes. Therefore, the application of MetaSPR chips for the real‐time monitoring of cellular activities represents a promising area for further exploration.

**FIGURE 1:**
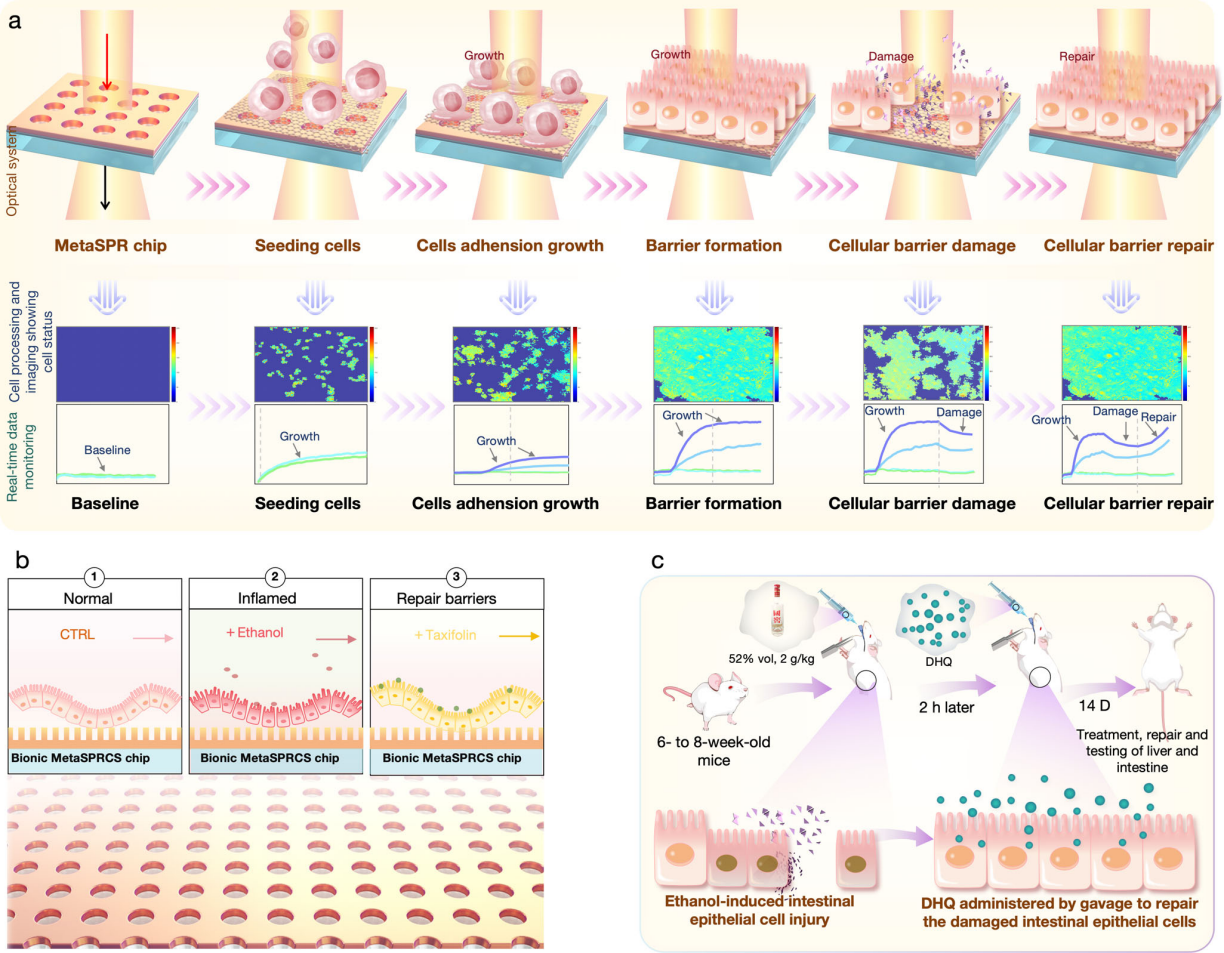
Schematic illustration of the development, validation, and applications of the metasurface plasmon resonance colorimetric sensor (MetaSPRCS) platform. (a) The platform facilitates real‐time monitoring of changes in cell states and imaging throughout the cell life cycle. (b) Application of the platform to assess alcohol‐induced intestinal damage. (c) Evaluation of dihydroquercetin's (DHQ) restorative effects on alcohol‐damaged intestinal cells, validated in vivo using mice.

In the study, we developed a nano‐plasmonic colorimetric chip system compatible with conventional incubators. This system uses a high Q‐factor resonant surface constructed with a nanocup array as an optical “stethoscope” and promotes rapid cell adhesion and monolayer barrier formation in Caco‐2 cells via a polyethyleneimine (PEI) interface. By integrating the retrained Segment Anything Model (SAM) into the real‐time imaging workflow, we tracked the entire process of cell adhesion, proliferation, barrier formation, ethanol damage, and repair at single‐cell resolution without labeling within 72 h. Specifically, we precisely controlled and fabricated a high‐performance and versatile metasurface plasmon resonance colorimetric sensor (MetaSPRCS) with a nanocup array structure using nanostructure design, high‐precision nanoimprint lithography, and electron beam evaporation technology. This sensor is suitable for visual imaging‐based monitoring and high‐throughput screening. We employed materials with high cell compatibility, such as PEI and type IV collagen, to modify the chip surface and fabricate a bionic cell chip that facilitates rapid cell attachment and supports viable cell growth. By integrating the MetaSPRCS chip with bright‐field imaging, we developed a cell‐state evaluation model based on the biomimetic cell chip. This MetaSPRCS cell‐on‐chip model supports continuous monitoring of dynamic changes in intestinal cells throughout their life cycle and simultaneously provides insights into the functionality of the intestinal barrier layer (Figure 1a).

Additionally, in vitro, we used the platform to evaluate the destructive effects of various liquors on the Caco‐2 enterocyte layer of human colon adenocarcinoma cells and the reparative effects of dihydroquercetin (DHQ) on alcohol‐damaged cells (Figure 1b). Platform accuracy and reliability were validated by comparing the results with those obtained from traditional Cell Counting Kit‐8 (CCK‐8) assays and crystal violet staining experiments. Simultaneously, we evaluated the reparative effects of DHQ on alcohol‐damaged intestinal barrier cells in mice and established a method for in vivo‐in vitro correlation assessment, which further validated the guiding relevance of the platform for in vitro evaluation (Figure 1c).

The MetaSPRCS platform allows for the monitoring of dynamic changes throughout the cell life cycle without interfering with cell behavior. The non‐cytotoxic nature of the operation and lack of fluorescent labeling address the limitations associated with long‐term cell monitoring and photobleaching. This work overcomes the long‐standing trade‐off between sensitivity, throughput, and cell‐friendliness in existing technologies, replacing the cumbersome traditional “multiple fixation‐staining‐endpoint reading” process with a scalable 96‐well chip, providing a real‐time, non‐destructive, and high‐throughput new paradigm for studying early barrier imbalances, screening regenerative food functional factors, and optimizing oral drug dosages.

## 2 Results and Discussion

### 2.1 Preparation and Performance Validation of the Visualized MetaSPRCS Chips

**FIGURE 2:**
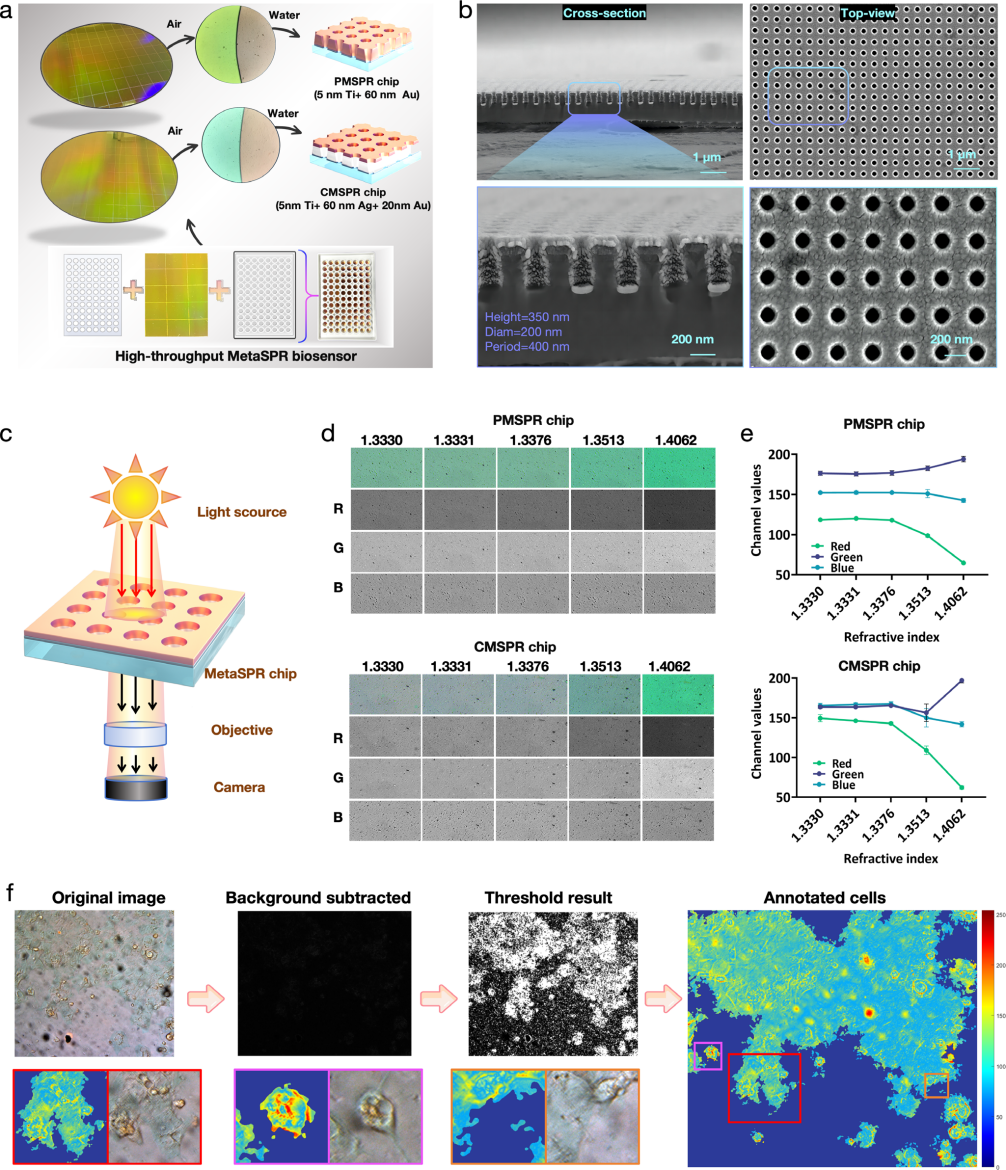
Development of a real‐time cell status monitoring model based on the MetaSPR chip (a) Photographs of the two types of metal‐based chips, accompanied by transmitted light microscopy images that illustrate the light scattering effects of the chips in air and water. (b) Scanning electron microscopy (SEM) images of the nanostructured arrays on the MetaSPR chip. (c) Schematic representation of optical components for the high‐throughput real‐time cell status monitoring model. (d) Comparison of color changes and RGB channel variations for two MetaSPR chips immersed in sucrose solution with different RIs: 1.3330 for deionized distilled water, 1.3331 for 0.078% sucrose, 1.3376 for 3% sucrose, 1.3513 for 12.5% sucrose, and 1.4062 for 50% sucrose. (e) Comparison of color changes and RGB channel variations for two MetaSPR chips immersed in sucrose aqueous solutions with different RIs. (f) Schematic diagram of the image processing method used for the imaging platform.

By leveraging the outcomes of the optimized simulation design, we successfully fabricated two types of MetaSPR chips using nanoimprint lithography and electron‐beam evaporation. The chips used in this study are a pure gold MetaSPR chip (PMSPR, 5 nm Ti+60 nm Au) and a gold‐silver composite chip (CMSPR, 5 nm Ti+60 nm Ag+20 nm Au) (Figure 2a). A primary advantage of the MetaSPR technology lies in its ability to precisely control light at the nanoscale [34]. Consequently, the chips exhibit a multicolor effect under natural light, attributable to light scattering by the nanostructured arrays. Notably, the PMSPR and CMSPR chips displayed distinct colors in air and water, indicating a high sensitivity to the surrounding refractive index (RI). Scanning electron microscopy images, both cross‐sectional and planar, revealed uniform nanocup arrays, validating the high consistency of the chip fabrication process (Figure 2b). These structural features suggest that the observed light‐scattering effects result from the interaction of visible light with the nanostructured arrays. The sensitivity of our plasmonic chips to RI changes was evaluated using glycerol solutions, demonstrating distinct performance characteristics of the PMSPR and CMSPR chips (Figure S1).

### 2.2 Selection of Chips for the Visualization MetaSPRCS Platform

We meticulously designed a bottomless conical 96‐well plate optimized for high‐throughput cell culture and integrated it with the two types of MetaSPR chips described above (Figure 2a). This setup leverages the principles of surface plasmon resonance, where the interaction between light and the metasurface structure leads to resonant phenomena that are highly sensitive to changes in the RI near the sensor surface. Additionally, by coupling these detection devices with a conventional bright‐field microscope or general optical system, we successfully constructed a MetaSPRCS imaging platform (Figure 2c). Using this platform, we tested a range of sucrose concentrations and observed that the chip surface color changed with variations in the RI (Figure 2d). By imaging the chip's response to different RIs and analyzing the red‐green‐blue (RGB) channels [35] using ImageJ software, we found that the changes in the red (R) channel for both chips exceeded those in the green and blue channels, with the PMSPR chip exhibiting more significant variations in the R channel than the CMSPR chip (Figure 2e). The PMSPR chip demonstrated superior transmittance and sensitivity, making it more suitable for the imaging platform, as detailed in Figure S2.

Based on the chip's response to RI changes, the PMSPR chip was identified as a more suitable option for the imaging platform. Additionally, protein interaction experiments confirmed its optimal SPR performance (Figure S3). Consequently, the PMSPR chip was selected as the substrate for the biomimetic chip. To investigate the optimal PMSPR chip thickness, an optical simulation of the system was performed using the Finite‐ Difference Time‐Domain (FDTD) software, which identified 5 nm Ti+50 nm Au as the optimal configuration (Figure S4).

### 2.3 Artificial Intelligence‐Enhanced Data Processing for Real‐Time Cell Monitoring on the MetaSPRCS Platform

We implemented an artificial‐intelligence‐enhanced, end‐to‐end pipeline that integrates a re‐trained SAM [36] with the MetaSPRCS platform to enable label‐free, real‐time monitoring of intestinal barrier dynamics. High‐resolution colorimetric images acquired by the integrated imaging platform are first fed into a SAM encoder–decoder stack that we fine‐tuned exclusively on MetaSPRCS micrographs. The model is prompted to segment the R‐channel background, whose mean intensity is then subtracted from the original frame so that the residual signal reflects only the plasmonic response arising from cells adhered to the metasurface. This background‐corrected image is subsequently re‐analyzed by the same SAM instance, now conditioned to delineate individual cellular footprints with sub‐micron fidelity. The resulting binary masks are converted into SPR‐active area and frame‐wise signal amplitude, and the corresponding intensity values are color‐mapped to produce spatial heat maps that evolve in register with cell attachment, spreading, and barrier modulation. Using this approach, we constructed a pipeline for segmenting cells and extracting SPR signal changes during cell growth on the MetaSPR chip (Figure 2f). This comprehensive data processing pipeline, augmented by artificial intelligence, enabled accurate, real‐time, and label‐free monitoring of dynamic cell behavior, providing a robust foundation for studying cellular responses to various stimuli.

### 2.4 Development of Cell‐Mimetic Chips and Establishment of Cell Life Cycle Monitoring Models Based on the MetaSPRCS Platform

We developed a label‐free, long‐term, and real‐time monitoring platform designed to track the complete life cycle of intestinal cells using a biomimetic chip. This innovative technology involves using MetaSPRCS imaging to detect changes in transmissivity resulting from cellular adhesion, proliferation, and apoptosis, facilitating continuous visualization of cellular states. To establish a robust monitoring model, we systematically optimized surface modifications and cell‐seeding parameters.

**FIGURE 3:**
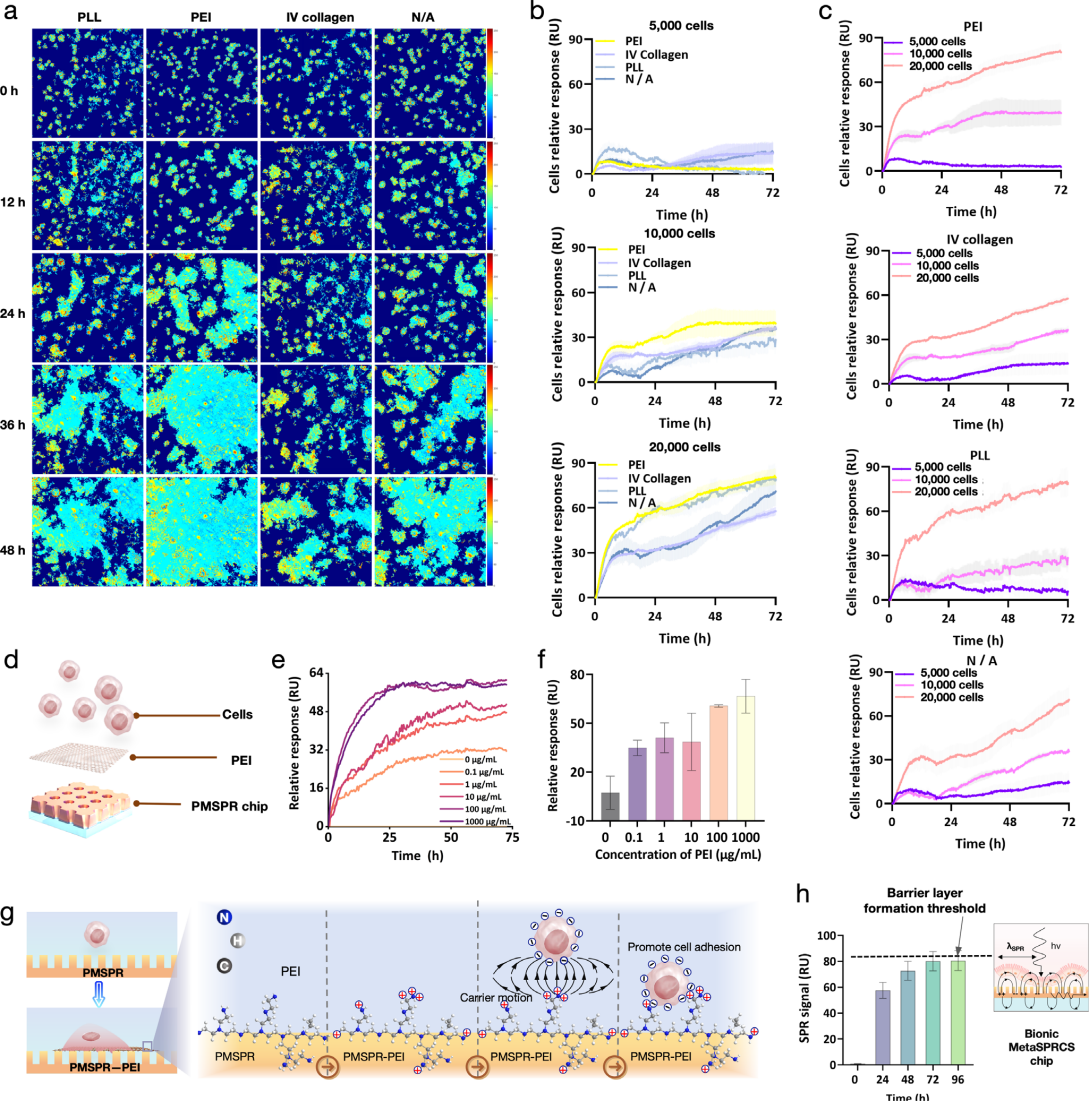
Label‐free real‐time monitoring of the intestinal cell life cycle using the MetaSPRCS platform (a) Images of cells (0, 12, 24, 36, and 48 h) subjected to different surface modifications (poly‐L‐lysine (PLL), polyethyleneimine (PEI), type IV collagen, and unmodified (N/A). (b) Comparison of signal intensity across varying cell densities (0.5, 1, and 2 w cells per well). (c) Real‐time dynamic curves illustrating cell growth under optimized conditions. (d) Final design of the biomimetic chip: 2 w cells/well on PEI‐modified chips. (e) Variations in the surface plasmon resonance (SPR) signal with different PEI concentrations (0–1000 µg/mL). (f) Optimal PEI concentration (100 µg/mL) determined based on signal consistency. (g) Schematic illustration of the underlying principle of cell adhesion on the biochip. (h) SPR signal threshold for barrier layer formation based on the biochip (left), schematic diagram of the changes in optical and plasmon resonance signals of bionic MetaSPRCS chips when forming cell barriers (right).

Our screening of surface modifications indicated that chips coated with PEI exhibited superior performance compared with those modified with poly‐L‐lysine (PLL), type IV collagen, and unmodified controls. Real‐time imaging revealed that PEI modification markedly enhanced cell adhesion efficiency across all monitored time points (Figure 3a and Figure S5 and Video S1). The cell inoculation density was optimized to achieve a balance between spatial resolution and signal robustness. At a density of 20,000 (2 w) cells per well, the platform produced a stable signal and captured the maximum signal associated with cell division, whereas lower densities (5,000–10,000 (0.5–1 w) cells per well yielded sparse signals (Figure 3b and Figure S6a). A comparison of the real‐time monitoring curves across different conditions indicated that the 2‐w cells per well yielded the most substantial real‐time monitoring signal (Figure 3c and Figure S6b). Furthermore, the PEI‐modified chip exhibited the strongest SPR signals (Figure 3d,e) and demonstrated no nonspecific signal across various cell comparison groups (Figure S6c). Therefore, our platform used PEI for cell immobilization (Figure 3d). To optimize monitoring efficacy, we further refined the PEI concentration and discovered a positive correlation between concentration and signal strength, with saturation occurring at 100 µg/mL (Figure 3e) and demonstrating the highest consistency (Figure 3f). Consequently, the optimal PEI concentration was established to be 100 µg/mL. A substantial change in the surface contact angle was observed before and after PEI modification (Figure S7). This change was attributed to surface bioactivation‐induced changes in the surface energy, indicating an enhanced adhesion and diffusion of the PEI substrate in aqueous solutions, promoting cell adhesion and spreading (Figure S8).

Based on a comparative analysis of surface functionalization strategies, PEI demonstrated superior performance in enhancing cell‐chip interfacial compatibility, substantially outperforming PLL, collagen IV, and unmodified substrates. The cationic properties of PEI enable electrostatic interaction with the negative charges on the cell surface, providing a stable substrate for cell attachment, promoting cell adhesion and growth (Figure 3g). Under the modification of PEI, a cell barrier layer rapidly forms on the surface of the bionic chip. By monitoring changes in SPR signals at different time intervals, it can be observed that as the monitoring time increases, the cell barrier layer gradually forms within 72 h, and the signal no longer increases after 72 h, indicating that the barrier layer has formed (Figure 3h). The SPR signal threshold for the barrier layer formation on the biochip was determined through repeated experiments. The experiments were repeated four times using chips from different batches, with each repeat comprising three replicates. The threshold was calculated as the average of all signals, ensuring result reliability and reproducibility (Figure S9). The SPR signal changes we observed reflect the overall integrity of the intestinal barrier, including the status of tight junctions. Our system's high sensitivity enables it to detect disruptions at the tight junction level, which are crucial for barrier function. The consistent saturation of the SPR signal at a specific threshold across multiple measurements indicates the formation of a complete barrier layer, corresponding to full cellular coverage, as verified by imaging. This feature allows for real‐time monitoring of barrier integrity and offers insights into the dynamic cellular changes.

We effectively established a dynamic monitoring model for studying the complete life cycle of intestinal wall cells using the biomimetic chip MetaSPRCS platform. This refined model facilitates continuous and noninvasive observation of cell cycle dynamics. In contrast to conventional endpoint assays, this model can be used to monitor real‐time alterations for approximately 72 h without the need for fluorescent labeling or destructive sampling methods.

### 2.5 Evaluation of Alcohol‐Induced Intestinal Barrier Damage Based on MetaSPRCS Modeling

**FIGURE 4:**
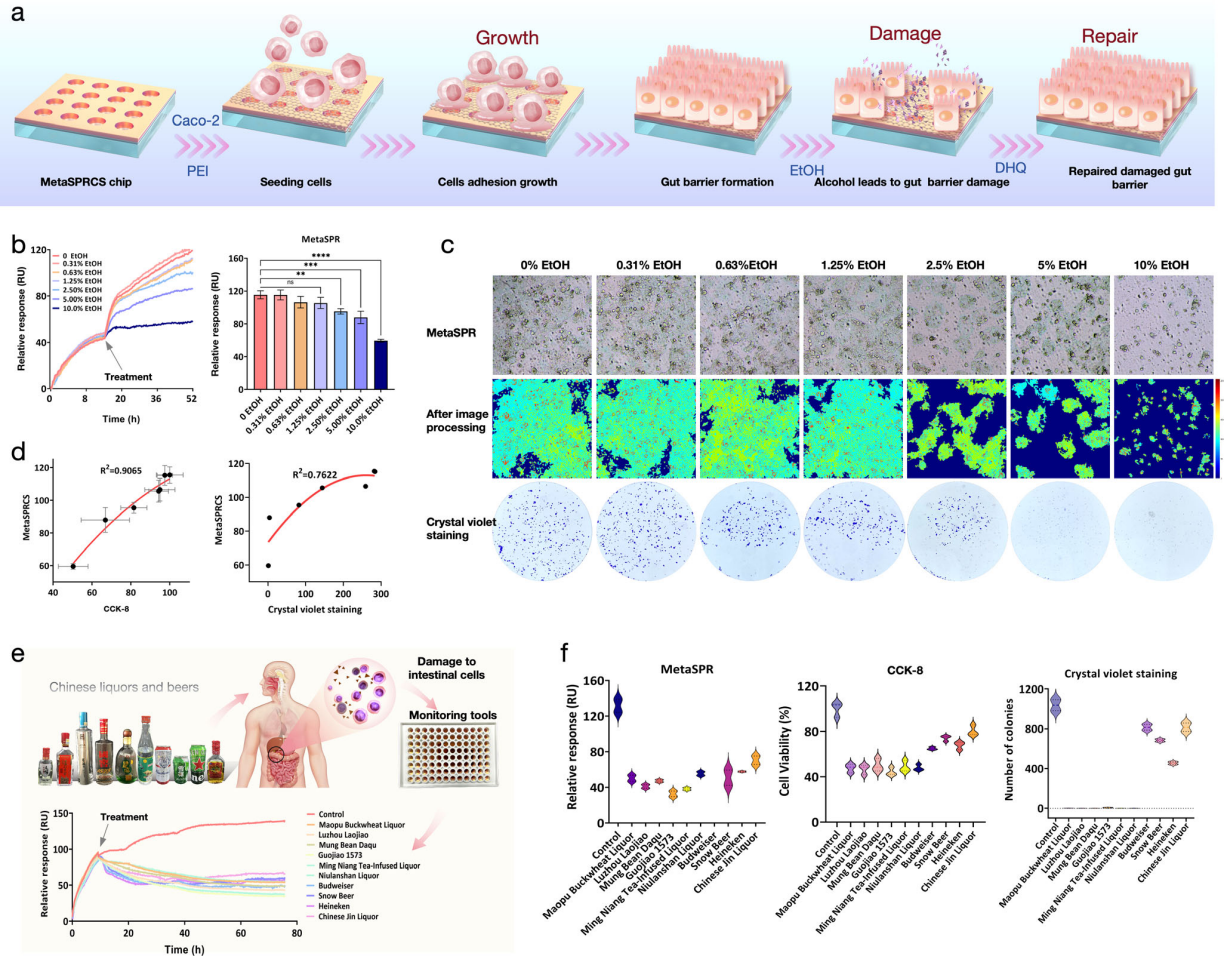
Assessment of cell barrier formation, damage, and repair on biomimetic chips. (a) Schematic workflow for assessing alcohol toxicity and cell barrier repair using the MetaSPRCS platform. (b) Real‐time assessment of intestinal cell injury under ethanol gradients (0.3%–10% v/v). (c) Evaluation of cell barrier formation on biomimetic chips and the effects of alcohol‐induced damage, with a comparison to crystal violet staining results. (d) Correlation analysis of cytotoxicity evaluations across three methods: MetaSPRCS, CCK‐8, and crystal violet. The CCK‐8 data represent the calculated cell survival rate. (e) MetaSPRCS‐based evaluation of the effects of Chinese liquors and beers on intestinal barrier integrity. (f) Ranking of liquor and beer cytotoxicity as determined through MetaSPRCS, CCK‐8, and crystal violet assays.

To validate the clinical utility of the MetaSPRCS monitoring model, we simulated the intestinal barrier using Caco‐2 cells as a representative example and systematically assessed ethanol‐induced damage based on the optimized MetaSPRCS scheme (Figure 4a). Real‐time dynamic monitoring revealed that ethyl alcohol (EtOH) induced concentration‐dependent cytotoxicity: the SPR signal changes in the 0.3%–1.25% EtOH‐treated groups were not statistically different from those in the control group (*p* ≥ 0.05). However, 2.5% EtOH triggered significant cytotoxicity (*p* < 0.01), and treatment with 10% EtOH caused a complete collapse of the SPR signals within 2 h (*p* < 0.0001), indicating rapid cell membrane breakdown (Figure 4b). The cellular status of the chip was monitored using the MetaSPRCS. When the biomimetic chip with an intestinal barrier was exposed to varying alcohol concentrations, distinct degrees of barrier damage were observed. Comparative analysis of the chip, processed, and crystal violet staining images revealed differences in cell barrier integrity. The processed images provided detailed insights into the cell barrier completeness. Consistent trends were observed across all three image types (Figure 4c), validating the reliability of the MetaSPRCS system in assessing intestinal barrier damage induced by different alcohol concentrations. Through parallel validation of metabolic activity (CCK‐8) and adherent cell mass (crystal violet staining), the MetaSPRCS assay results were highly consistent with those obtained from traditional methods (CCK‐8: R^2^ = 0.9065; crystal violet: R^2^ = 0.7622) (Figure 4d). The kinetic patterns observed with the MetaSPRCS platform were closely correlated with the traditional endpoint assay results, demonstrating the reliability and accuracy of MetaSPR technology. Notably, our model platform could monitor the cytotoxic effects of 2.5% EtOH on intestinal wall cells, whereas the CCK‐8 assay could only detect effects at 5% EtOH, highlighting the high sensitivity of the MetaSPRCS platform. This sensitivity was further illustrated by parallel measurements of the metabolic activity and adherent cell mass (Figure S10).

To investigate the risk of intestinal injury in real drinking scenarios, we further evaluated the cytotoxicity of 10 commercially available liquors (ethanol concentration 38%–52% v/v) and beer (ethanol concentration 2.5%–4.7% v/v). Caco‐2 cells were cultured on the chip surface. After monitoring their growth for 12 h, different brands of liquors and beers, diluted 1:1 with culture medium, were introduced to observe real‐time changes in cell status. A comparative analysis of Chinese liquors revealed alterations in toxicity that extended beyond the ethanol content (Figure 4e). MetaSPRCS monitoring indicated considerable brand‐specific alcohol toxicity: Guojiao 1573 induced the most pronounced cellular damage, which was significantly greater than that caused by Niulanshan and Luzhou Laojiao (Figure 4f). Additionally, crystal violet staining confirmed that the observed differences were attributed to the destruction of the cell monolayer caused by Guojiao 1573 treatment, whereas the other liquors caused only localized detachment (Figure S11). Furthermore, liquor induced significantly greater cytotoxicity than beer, with Chinese liquor, a relatively mild variant, causing the least damage among the ten liquors tested.

Methodological comparisons revealed that the MetaSPRCS platform could detect early barrier damage signals within 30 min, representing a 12‐fold improvement in timeliness compared with the CCK‐8 method (>6 h) and ≥36‐fold improvement over crystal violet staining (>48 h). This highlights the rapid, highly sensitive, and high‐throughput capabilities of our model platform. The consistency of the evaluation results from the three methods (CCK‐8: R^2^ = 0.9020; crystal violet: R^2^ = 0.7403) further validates the accuracy and reliability of the platform (Figure S12).

### 2.6 Evaluation of DHQ's Restorative Effects on Alcohol‐Induced Intestinal Injury Using the MetaSPRCS Platform

**FIGURE 5:**
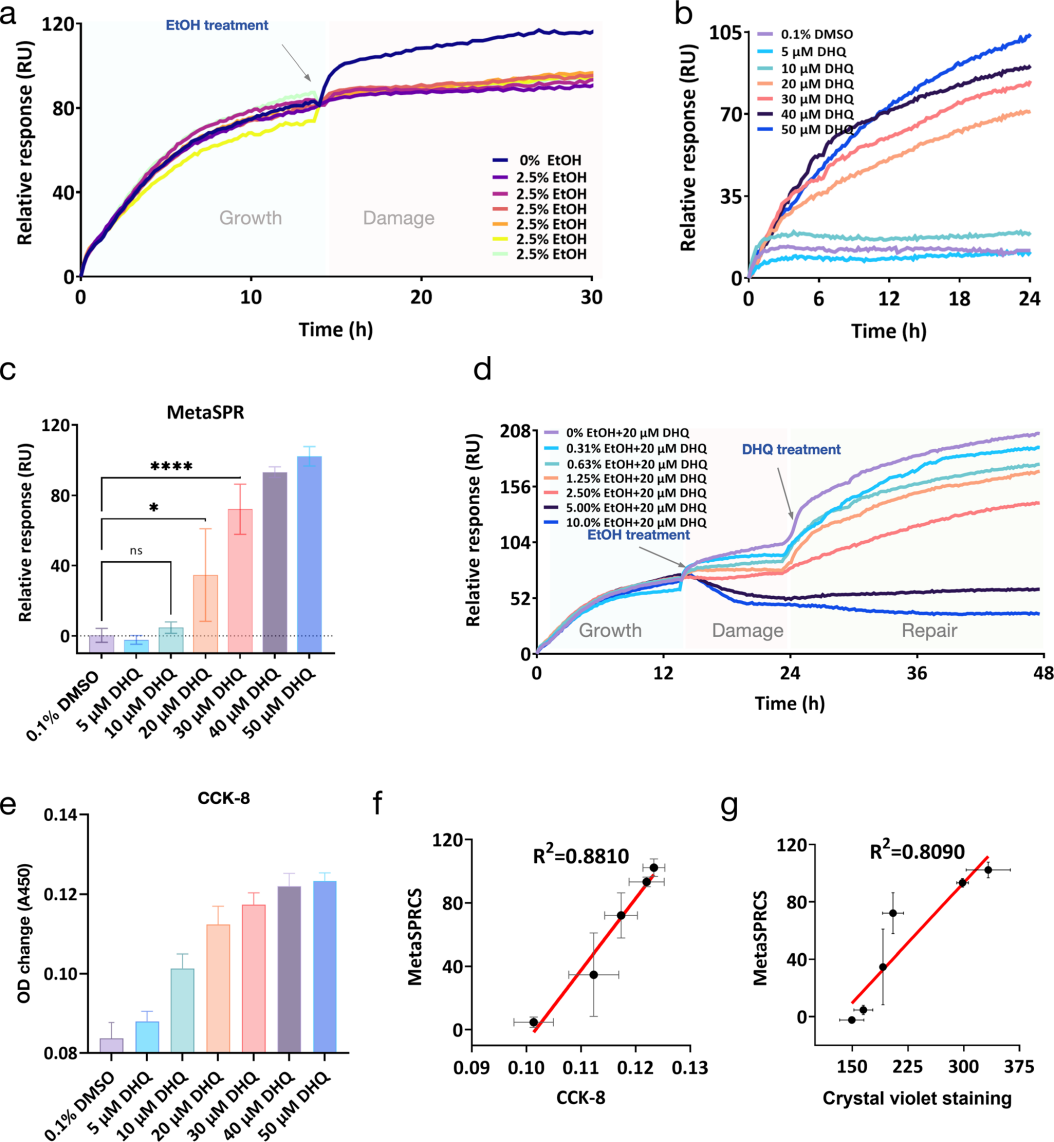
Evaluation of the restorative effects of DHQ. (a) Real‐time monitoring of Caco‐2 cell viability under alcohol exposure (2.5% v/v ethanol). (b) Restoration kinetics of alcohol‐injured cells treated with dihydroquercetin (DHQ) (5–50 µm). (c) Quantitative analysis of DHQ concentration‐dependent repair efficacy (ns: not significant; *: *p* < 0.05, ****: *p* < 0.0001). (d) Alcohol dose‐response rescue assay with fixed‐dose DHQ (20 µm). (e) CCK‐8 validation of DHQ‐mediated repair. (f) Correlation analysis between CCK‐8 and MetaSPRCS readouts (R^2^ = 0.8810). (g) Correlation analysis between crystal violet and MetaSPRCS data (R^2^ = 0.8090).

To further assess the platform's interaction monitoring efficiency, we established a damage‐repair model to evaluate its capability for monitoring intestinal barrier recovery in response to novel food ingredients. Although DHQ promotes intestinal barrier repair, data regarding its real‐time and dose‐dependent effects remain limited [37]. Therefore, we used this novel food ingredient in a cell‐based sensing platform to investigate its dynamic interactions with the intestinal barrier. The MetaSPRCS platform was used to assess the DHQ‐mediated repair of alcohol‐damaged intestinal cells, with validation provided by the results of conventional assays (CCK‐8 and crystal violet staining) and animal experiments. Alcohol‐induced cell injury (2.5% v/v ethanol) generated highly reproducible real‐time damage curves across multiple chip wells, demonstrating excellent chip‐to‐chip consistency (Figure 5a). Dose‐response analysis revealed DHQ's concentration‐dependent restorative effects: 5–10 µm DHQ failed to reverse alcohol‐induced damage, whereas 20 µm caused partial recovery (*p* < 0.05), and 30–50 µm achieved significant restoration (*p* < 0.0001) (Figure 5b,c).

To delineate DHQ's therapeutic window, we challenged Caco‐2 cells with varying ethanol concentrations (0.3%–10% v/v) followed by 20 µm DHQ treatment. Real‐time dynamic curves demonstrated dose‐limited reparative capacity: 0.3%–2.5% alcohol‐induced damage was reversible, while injuries caused by 5%–10% v/v ethanol exceeded DHQ's rescue potential (Figure 5d). Bidirectional validation—fixed ethanol (2.5% v/v) with variable DHQ (10–50 µm) vs fixed DHQ (20 µm) with variable ethanol (0.3%–10% v/v)—confirmed DHQ's efficacy threshold (>20 µm) and alcohol toxicity limits (<5% v/v) for functional repair. These findings underscore the capability of the MetaSPRCS platform for label‐free longitudinal monitoring of cellular responses across diverse physiological states. The ability to conduct real‐time intuitive analyses of the effects of components on cellular dynamics online can provide extensive information for reference. This is more objective and supportive than the experimental results provided by methods such as MTT, which can only provide results at a specific point.

Parallel validation using CCK‐8 and crystal violet staining showed strong concordance with the MetaSPRCS data (Figure 5e). The Pearson correlation coefficients reached 0.8810 (CCK‐8) and 0.8090 (crystal violet), confirming methodological consistency (Figures 5f,g and Figure S13). Notably, traditional endpoint assays require cell fixation/staining (inherently cytotoxic), whereas our platform enables non‐destructive, real‐time tracking over extended durations while maintaining cell viability. This contrast highlights the practical advantages of the MetaSPRCS in preserving native cell physiology during dynamic therapeutic evaluation. Moreover, our detection system is compatible with standard incubators, operates electrode‐free, is suitable for high‐throughput imaging, and enables label‐free monitoring. These features collectively enhance its applicability and efficiency compared to traditional methods.

### 2.7 Animal Experimentation and Establishment of In Vivo‐In Vitro Correlation

**FIGURE 6:**
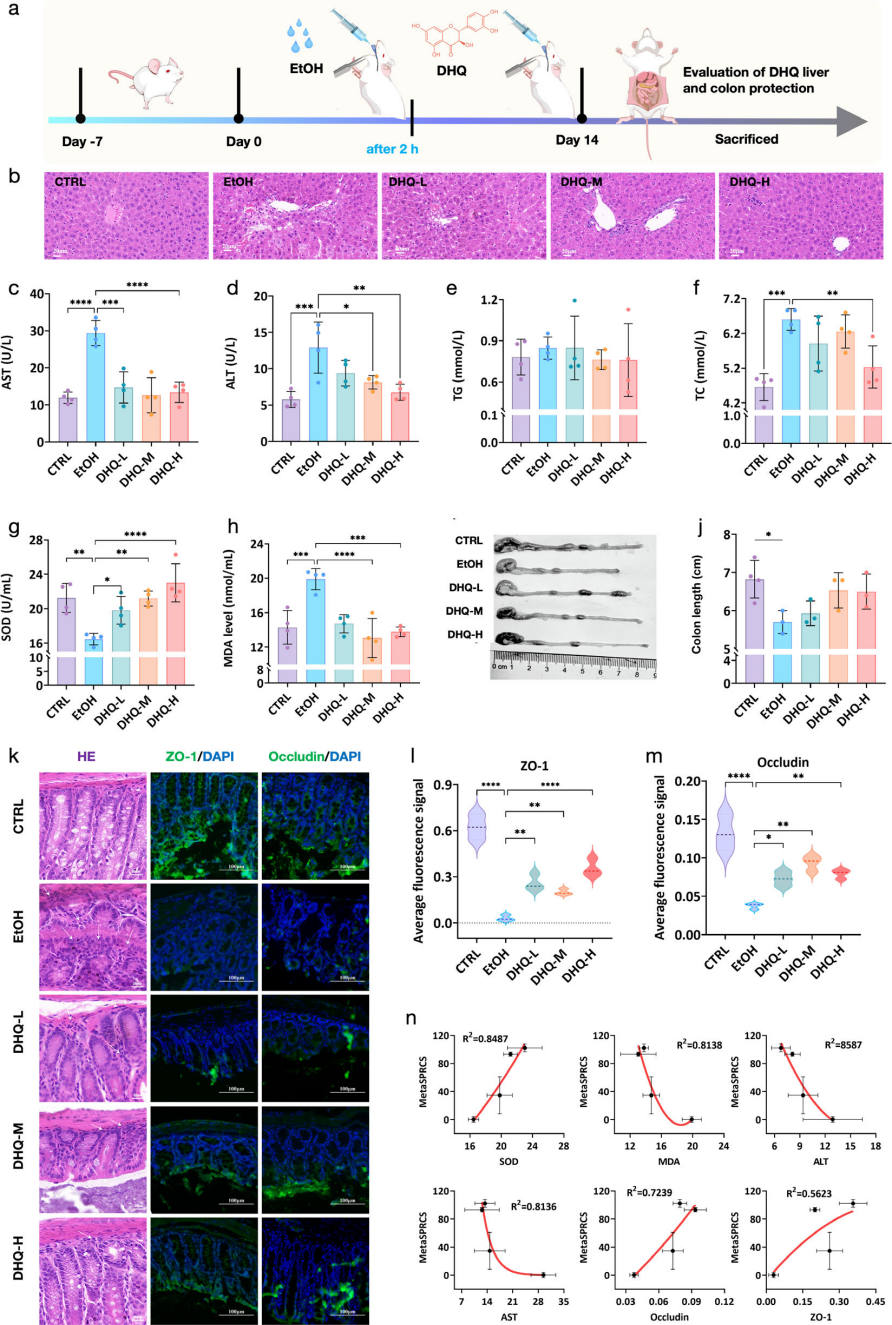
In vivo validation of the repair of alcohol‐induced liver and intestinal injuries using different DHQ doses. (a) Timeline for the establishment of the alcohol‐induced liver and intestinal injury model in mice and the intervention of DHQ. (b) Hematoxylin and eosin (H&E) staining of liver tissue. (c,d) Effect of DHQ on levels of AST (c) and ALT (d) in the serum of mice. (e,f) Effect of DHQ on levels of TG (e) and TC (f) in the serum of mice. (g–h) Effect of DHQ on liver SOD (g), and MDA (h) in alcohol‐induced mice. (i,j) The length of the colon of the five treatment groups. (k) Representative image of H&E staining and immunofluorescence image of ZO‐1 and Occludin in colon tissue of the four treatment groups (detailed view). (l) Average fluorescence signal of the ZO‐1 in the five treatment groups. (m) Average fluorescence signal of the Occludin in the five treatment groups. (n) Correlation analysis of the reparative effects assessed using the MetaSPRCS platform and those evaluated using various indicators in animal experiments.

To correlate the MetaSPRCS platform with in vivo effects, we performed correlation analyses between the in vitro system and animal experiments to validate its accuracy and broader applicability. We evaluated varying DHQ doses for repairing alcohol‐induced damage in vivo. After a 7‐day acclimation, C57BL/6J mice were orally administered Guojiao 1573 (52% v/v, 2 g/kg) on day 0. After 2 h, DHQ was administered orally at 20, 40, or 80 mg/kg. This protocol was followed for 14 consecutive days (Figure 6a). Throughout the experimental period, mice body weight and hepatic index were monitored. The control group (CTRL) exhibited a gradual increase in average body weight (30.05 ± 1.49 g), whereas the alcohol‐treated group (EtOH) demonstrated a decreasing trend (27.47 ± 0.47 g) (Figure S14a). Mice treated with DHQ showed a significant increase in body weight and a reduced hepatic index (3.78 ± 0.147%) compared with the EtOH group (4.05 ± 0.272%) (Figure S14b). We evaluated the hepatic histopathological changes using liver hematoxylin and eosin (H&E) staining and scored the sections using the Knodell scoring system (Figure 6b and Figure S14c). DHQ alleviated liver enlargement and congestion in alcoholic liver disease (ALD) mice. H&E staining revealed well‐organized hepatocytes in the CTRL mice, whereas the mice in the EtOH group showed pericentral cytoplasmic vacuolation, indistinct cell boundaries, inflammatory infiltration, and localized hepatocyte necrosis. Conversely, DHQ‐treated mice exhibited lower tissue scores, suggesting that DHQ ameliorated these pathological alterations in a concentration‐dependent manner.

To assess the effect of DHQ on liver function and lipid profiles in mice, we measured the serum alanine transaminase (ALT) and aspartate transaminase (AST), triglycerides (TG), and total cholesterol (TC). Alcohol administration significantly increased serum ALT, AST (*p* < 0.01), and TC (*p* < 0.01) levels, while TG levels showed a slight elevation compared with the control group (Figure 6c–f), collectively indicating alchol‐liver damage. Treatment with 20, 40, and 80 mg/kg DHQ reversed these effects, demonstrating its efficacy in alleviating alcohol‐induced hepatotoxicity. To evaluate the antioxidant effect of DHQ on mice after alcohol exposure, we measured serum superoxide dismutase (SOD) levels and malondialdehyde (MDA) content. Ethanol‐treated mice showed significantly reduced SOD activity, which was restored by DHQ in a dose‐dependent manner (Figure 6g), while MDA levels were markedly elevated, indicating oxidative stress. DHQ treatment significantly lowered the MDA levels in a dose‐dependent manner (Figure 6h). These results suggest that DHQ mitigates oxidative damage by enhancing SOD activity and reducing MDA levels, effectively counteracting alcohol‐induced oxidative stress.

Compared with controls (6.825 ± 0.49 cm), EtOH‐treated mice showed significant shortening of colon length (5.7 ± 0.3 cm, *p* < 0.05) (Figure 6i). DHQ treatment reversed this effect in a dose‐dependent manner. H&E staining of the colon revealed distorted crypts, inflammatory infiltration, and damaged intestinal epithelium in the EtOH group. DHQ treatment mitigated the intestinal inflammation and tissue damage (Figure 6j). In addition to its anti‐inflammatory effects, DHQ induced higher expression of tight junction (TJ) proteins (ZO‐1 and occludin) in the spinous and granular layers of the mucosa than in the EtOH group (Figures 6k–m and Figure S15). DHQ effectively alleviates colon tissue damage by alleviating inflammation and enhancing gut barrier integrity. These findings align with previous reports [38] indicating that DHQ significantly upregulates the expression of key tight junction (TJ) proteins, including claudin‐1, ZO‐1, and occludin, at both transcriptional and translational levels. Furthermore, DHQ helps maintain the continuous and organized distribution of these proteins along the cell membrane, which is essential for restoring paracellular barrier integrity. Besides, DHQ is known to possess strong antioxidant properties, which may contribute to barrier protection by reducing oxidative stress‐induced damage to epithelial cells and TJ proteins. Reports have indicated that DHQ exerts potent anti‐inflammatory effects by inhibiting the activation of the NF‐κB pathway. By attenuating the inflammatory milieu, DHQ creates a favorable environment for barrier repair and reduces cytokine‐induced TJ disruption.

The integration of in vitro MetaSPRCS‐based evaluation with in vivo animal validation establishes a translational bridge for assessing the therapeutic potential of DHQ. The MetaSPRCS platform revealed critical thresholds for DHQ efficacy (>20 µm) and alcohol toxicity (<5% v/v EtOH), which informed the dose selection in murine studies (20–80 mg/kg DHQ). This cross‐scale consistency (Figure 6n) is evident in DHQ's concentration‐dependent repair effects: both systems demonstrated dose‐responsive restoration of barrier integrity, with 20 µm DHQ initiating partial recovery in vitro and 20 mg/kg DHQ alleviating colon shortening in vivo. The platform's ability to resolve sub‐lethal alcohol injury (0.3%–2.5% v/v EtOH) and real‐time repair kinetics aligns with in vivo observations of TJ protein upregulation (ZO‐1/occludin) and oxidative stress mitigation (SOD↑/MDA↓). Notably, MetaSPRCS‐detected early‐phase cytoskeletal stabilization (within hours) preceded histological improvements in the colon tissue (day‐long recovery), suggesting that the platform captures the initial events of mucosal repair. Although traditional assays (CCK‐8 and crystal violet) have confirmed endpoint correlations (R^2^ > 0.9), they cannot track dynamic repair trajectories. For instance, MetaSPRCS revealed DHQ's rapid intervention, whereas animal models were used to quantify systemic outcomes (14‐day hepatic/colonic recovery). This complementary temporal resolution highlights the utility of the MetaSPRCS in predictive therapeutic profiling, particularly for optimizing dosing regimens. The translational relevance is further supported by shared biomarkers: MetaSPR spectral shifts (reflecting barrier integrity) correlated with in vivo TJ protein expression, whereas alcohol's dual toxicity (intestinal barrier disruption and hepatic oxidative stress) was consistently mitigated by DHQ in both models. However, the physiological complexity necessitates caution; the platform's 2D model could not fully recapitulate systemic alcohol effects (e.g., 52% v/v ethanol in vivo vs. 10% v/v in vitro thresholds), underscoring the value of combined *in vitro‐in vivo* approaches.

To establish a correlation between in vivo alcohol levels and in vitro concentrations, we determined the therapeutic threshold of DHQ in both settings. Our in vitro studies identified a functional repair threshold at 20 µm DHQ, with higher concentrations demonstrating significant barrier repair efficacy. In our in vivo mice model, DHQ doses ranging from 20 to 80 mg/kg effectively mitigated alcohol‐induced damage. Specifically, 20 mg/kg DHQ improved colon length, enhanced tight junction protein expression, and reduced oxidative stress markers. Animal experiments confirmed the predictive capability of our *in vitro‐to‐in vivo* dose conversion for DHQ, providing a promising basis for the administration of novel cell‐repairing food ingredients. This study thus offers a high‐throughput, non‐destructive tool for assessing intestinal homeostasis and establishing a preliminary dose conversion relationship between in vitro and in vivo settings.

## 3 Conclusions

In conclusion, in the present study, we successfully established and validated a MetaSPR biomimetic chip‐based model for real‐time assessment of the intestinal cell barrier. This model is distinguished by its capacity to facilitate real‐time, label‐free, and prolonged observation of barrier cell dynamics, representing a notable advancement in cell barrier studies. Through several comparative experiments conducted under diverse conditions, gold‐coated chips were observed to enhance the adhesion and proliferation of Caco‐2 cells, indicating that the material characteristics of the chip surface substantially influence cellular behavior. Additionally, PEI was recognized as an effective agent for accelerating cell adhesion and growth, suggesting its potential utility as a beneficial additive in cell culture systems aimed at improving cell attachment. Furthermore, a cell seeding density of 20 000 cells per well was established as optimal for the MetaSPR microplate detection platform, highlighting the critical role of cell density in obtaining accurate and reliable monitoring outcomes.

In this study, we developed a real‐time, label‐free, high‐throughput evaluation system based on a MetaSPRCS platform. By integrating nanophotonic metasurfaces with adaptive imaging algorithms, we achieved label‐free, continuous observation of intestinal barrier dynamics at the single‐cell level while maintaining cellular viability during 72 h of monitoring—an achievement that surpasses the capabilities of fluorescence‐based techniques. The dual functionality of the platform quantifies alcohol‐induced epithelial damage at sub‐lethal concentrations (2.5% EtOH) and facilitates real‐time assessment of therapeutic interventions, exemplified by DHQ barrier‐restorative effects.

This study advances the field by developing an RI‐sensitive nanocup array architecture that captures multidimensional cellular responses, including adhesion, apoptosis, and membrane remodeling, via integrated spectral signatures. Cross‐model validation further bridged in vitro mechanisms with in vivo therapeutic outcomes, addressing the persistent translational gap in biomedicine. Together, this integrated platform establishes a sensitive and dynamic tool for probing cell barrier dynamics and provides a foundation for guiding the dosage application of new food ingredients. Its label‐free, real‐time monitoring of the Caco‐2 barrier layer also provides a foundation for extending this technology to diverse intestinal cell types within engineered intestinal organoids.

While the in vitro MetaSPRCS platform provides high‐resolution, real‐time monitoring of cellular responses, its direct translation to in vivo effects is limited by differences in physiological complexity and pharmacokinetics. Although precise dose conversion necessitates further refinement through pharmacokinetic studies, our in vivo mice model has confirmed the therapeutic potential of DHQ, with dose‐dependent repair effects correlating well with our in vitro findings. Future research will prioritize the development of more sophisticated in vitro models and detailed in vivo pharmacological analyses to enhance translational relevance. Additionally, future studies will focus on detailed concentration profiling and correlation analysis to establish robust models linking in vivo and in vitro data.

## 4 Experimental Section

### 4.1 Materials

Detailed experimental material information can be found in the Supporting Information.

### 4.2 Fabrication and Characterization of MetaSPR Biosensor

The fabrication of the MetaSPR biosensor utilized the established nanofabrication methodology developed by the research group [39, 40, 41]. A quartz substrate was patterned with a periodic tapered nanopillar array (top diameter: 100 nm, base diameter: 200 nm, height: 500 nm) using laser interference lithography to create a master mold. After applying a silane‐based hydrophobic treatment to the mold, UV‐curable adhesive was uniformly coated and covered with a PET film. The assembly underwent 45 s of UV curing at an intensity of 105 mW cm^−2^ before carefully demolding to transfer the inverse nanocup array structure onto the PET substrate. Finally, sequential deposition of plasmonic metal layers (5 nm Ti adhesion layer and 60 nm Au) was performed using electron beam evaporation to complete the biosensor architecture. The sheet was then divided into sections measuring 13×8.5 cm and secured to an open‐bottom 96‐well plate, thereby creating a 96‐well biosensor plate [42].

### 4.3 Comprehensive Performance Evaluation of MetaSPR Biosensor Chips

The SPR microimaging platform evaluates chip performance by analyzing the transmission response of light to varying RI changes. Pairs of chips were affixed to a bottomless 96‐well plate, creating a 96‐well chip plate. The chips were rinsed with 150 µL of ultrapure water, and the water on the chip surface was shaken off, this process was repeated twice. The color of the chip in the air was observed under an inverted microscope and documented through photography. Sucrose solutions with concentration gradients of 0%, 0.78125%, 3.125%, 12.5%, and 50% were prepared. The color of the chip was observed and photographed sequentially under the inverted microscope, progressing from the lowest to the highest concentration.

#### 4.3.1 Deep‐Learning‐Based Segmentation and SPR Signal Extraction on MetaSPRCS Biochips

All image processing was performed with a customised implementation of the SAM embedded in Cellpose‐SAM (cellpose v3.0) [43]. To adapt SAM to the low‐contrast, colorimetric signatures of MetaSPRCS micrographs, we adopted a human‐in‐the‐loop refinement protocol. 800 regions of interest were manually annotated by two independent operators using the Cellpose GUI; discrepancies were resolved by a senior microscopist. These annotations were used to fine‐tune the generalist Cellpose‐SAM checkpoint with default augmentations. High‐resolution colorimetric images were acquired every 1 h via the MetaSPRCS imaging platform. Each image was split into its R channel, which carries the dominant plasmonic information. The fine‐tuned SAM model was prompted with a single background point and segmented the non‐cellular regions. The mean intensity of this mask was subtracted from the original frame to remove substrate drift and illumination artefacts. The background‐corrected image was re‐submitted to the SAM instance to yield cell masks of individual cells with sub‐micron edge precision. Pixels enclosed by the masks were converted to SPR response units using a chip‐specific calibration curve. SPR‐active area and mean signal amplitude were computed frame‐to‐frame; spatial heat maps were generated by colour‐coding the intensity values.

### 4.4 Development of a Real‐Time Cell State Evaluation Model Utilizing MetaSPR Chips

#### 4.4.1 Cell Culture

Caco‐2 cells (RRID: CVCL_0025) were cultured in Dulbecco's modified Eagle's medium (DMEM) supplemented with 10% (v/v) fetal bovine serum (FBS) and 1% (v/v) penicillin–streptomycin. Cells were seeded at 2 × 10^6^ cells per 10 mL and maintained at 37°C in a humidified incubator with 5% CO_2_.

#### 4.4.2 Optimization of Growth Conditions

Three extracellular matrix solutions—PLL at a concentration of 100 µg/mL, PEI at 100 µg/mL, and collagen type IV at 100 µg/mL—were applied to 96‐well biosensor plates at a volume of 50 µL per well. Following a 2‐h incubation at 37°C, the plates were subjected to two washing cycles with ddH_2_O. Caco‐2 cells were then seeded into the pre‐coated plates at standardized densities (100 µL medium per well, with cell counts of 5,000, 10,000, and 20,000 cells per well). The dynamics of cellular adhesion and proliferation under varying substrate conditions were continuously monitored over a period of 72 h using an automated detection instrument, the WeSPR^TM^ Cell Analyzer (XLement, China), with scanning intervals of 15 min.

#### 4.4.3 Cell Adhesion and Proliferation

Caco‐2 cells were plated in 96‐well biosensor plates containing 100 µL of DMEM per well. The dynamics of cell attachment and proliferation were monitored at 15‐min intervals utilizing the cell analyzer. The duration of monitoring was adjusted between 24 and 72 h according to the specific requirements of the experiments.

### 4.5 Evaluation of Ethanol‐Induced Intestinal Barrier Disruption

Caco‐2 cells were seeded on 96‐well biosensor plates at a density of 2 × 10^4^ cells/well per 100 µL. Cellular dynamics were assessed in real‐time utilizing the cell analyzer that conducted scans at 15‐min intervals. After a 12‐h incubation period, during which the cells were in the logarithmic growth phase, ethanol solutions at varying concentrations (0%, 0.3%, 0.6%, 1.25%, 2.5%, 5%, and 10% wt.) were introduced. Continuous monitoring was then carried out to evaluate the extent of ethanol‐induced intestinal barrier impairment.

Real‐time cellular alterations were captured simultaneously using an imaging system. The data were subsequently processed by analyzing changes in R‐channel values, which were used to generate real‐time growth curves that illustrated the variations in SPR readings as the cellular life cycle progressed.

### 4.6 Assessment of DHQ's Impact on Intestinal Barrier Restoration

Caco‐2 cells were cultured in 96‐well biosensor plates, with each well containing 100 µL of medium at a standardized density of 20 000 cells per well. Real‐time cellular dynamics were observed using a cell analyzer, which conducted scans at 15‐min intervals. Following a 12‐h cultivation period, during which the cells were in the logarithmic growth phase, a 2.5% (wt.) ethanol solution was introduced, and cellular status was continuously monitored for an additional 12 h. After the ethanol‐induced disruption of the intestinal barrier, the treatment medium was substituted with fresh medium containing varying concentrations of DHQ (0, 5, 10, 20, 30, 40, and 50 µM). A subsequent 24‐h period of real‐time monitoring was conducted to quantitatively evaluate the restorative effects of the compound on the dysfunction of the intestinal barrier induced by ethanol.

### 4.7 Animal Experiment Design

Male C57BL/6 mice, 6–8 weeks old (body weight 22 ± 2 g), were obtained from Nanjing Anno Kang Co., Ltd. (Nanjing, China). All animal experiments were conducted in accordance with internationally recognized guidelines for the care and use of laboratory animals and were approved by the Animal Ethics Committee of Nanjing Normal University (approval number: IACUC‐20220246; approval date: December 30, 2022). Dihydroquercetin (DHQ, purity ≥ 98%) was obtained from Shanghai Macklin Biochemical Technology Co., Ltd. Guojiao 1573 (52% vol) was provided by Luzhou Laojiao in Sichuan. Mice were randomly divided into five groups (*n* = 4 per group): control group, model group, low‐dose DHQ group (model + DHQ 20 mg/kg), medium‐dose DHQ group (model + DHQ 40 mg/kg), and high‐dose DHQ group (model + DHQ 80 mg/kg). Mice were housed in a controlled environment (temperature: 25°C ± 2°C; humidity: 40%–80%) with a 12‐h light/dark cycle and had free access to food and water. The control group was orally administered with physiological saline. The alcohol group was orally administered with Guojiao 1573 (alcohol concentration: 52% vol, 2 g/kg). The three DHQ groups were orally administered with alcohol to ensure complete absorption of alcohol components, followed by DHQ at 20, 40, and 80 mg/kg, respectively. Oral administration was conducted for two weeks. After the final administration, mice were fasting for 24 h and then anesthetized with an intraperitoneal injection of sodium pentobarbital (70 mg/kg, Shanghai B Zoe Biotechnology Co., Ltd., Shanghai, China). Blood samples were immediately collected from the eyeball, left at room temperature for 40 min, and then centrifuged at 3000 g for 10 min at 4°C to obtain the supernatant for biochemical analysis. Anesthetized mice were euthanized by dislocation, and the liver and colon tissues were immediately dissected. All dissected mice carcasses were sent to the Experimental Animal Center of Nanjing Normal University for unified disposal. Liver sections were fixed in 4% paraformaldehyde, colon tissues were preserved in Carnoy's fixative, and the remaining liver and colon segments were stored at −80°C for further experiments.

### 4.8 Biochemical Analysis

Serum levels of ALT, AST, TG, and TC in mice were measured using ALT, AST, TG, and TC activity assay kits (Elabscience, Wuhan) with an automated biochemical analyzer (Hitachi 7020, Japan). Oxidative stress in mouse serum was assessed using MDA and SOD assay kits (Elabscience, Wuhan). All experiments were conducted according to the manufacturers' instructions.

### 4.9 Statistical Analysis

SPR imaging data were analyzed with Image‐Pro Plus 6.0 (Media Cybernetics, USA). Real‐time binding traces were processed in the XLement Data Analysis Suite (v5.3.0) to extract kinetic parameters via global fitting. MetaSPR datasets were analyzed with Origin 2021 (OriginLab, USA). All quantitative results are reported as mean ± standard deviation (SD) from at least three independent biological replicates (*n* = 3). All statistical analyses were performed in GraphPad Prism 9.5.1 (GraphPad Software, USA). A two‐sided *p*‐value < 0.05 was considered statistically significant (*p < 0.05, **p < 0.01, ***p < 0.001, ****p < 0.0001). Calibration curves were fitted with a four‐parameter logistic regression model to ensure accurate quantification.

## Author Contributions

Y.C., W.L., J.F., Y.S., and Y.Y. contributed equally to this article. Y.C. designed, simulated, fabricated the MetaSPR chip, performed its SEM characterization and contact angle measurements, established a biomimetic chip imaging system, collected experimental data on killing and repair using the chip, drafted the Results and Discussion sections, and revised the manuscript. W.L. cultured the cells, provided cell cultivation methods on the chip, performed crystal violet staining and CCK–8 experiments, and drafted the initial Methods and Introduction sections. J.F. performed animal experiments, analyzed the data, and drafted the initial Animal Experiment section. Y.S. took photos of the imaging chip, performed RI characterization, and drafted the initial Methods section. Y.Y. counted the crystal violet staining results and built an AI model to process the image signals of SPR imaging. Y.W. characterized the chip's transmittance, and drew PEI modification. R.L., H.Z., X.Y., and M.C. provided methodological support. Y.G. assisted with mice gavage. W.H., G.L.L., and Y.L. provided financial support. L.H. revised the initial manuscript and funding support. L.H. and Y.L. conceived and supervised the entire project.

## Conflicts of Interest

The authors declare no conflicts of interest.

## Supporting information




**Supporting File**: advs74084‐sup‐0001‐SuppMat.pdf.

**Supporting File**: advs74084‐sup‐0002‐VideoS1.mp4.

## Data Availability

The data that support the findings of this study are available from the corresponding author upon reasonable request.
