# Supplementary material for: Innovative Metasurface Colorimetric Cell‐on‐a‐Chip Sensor for Continuous, Label‐Free, Non‐Destructive Assessment of Intestinal Barrier Dynamics
Source: Adv Sci (Weinh). 2026 Jan 28;13(19):e23118. doi: 10.1002/advs.202523118 (PMC13045419; doi:10.1002/advs.202523118)
Supplement: Supplementary file 1 — Supporting File: advs74084‐sup‐0001‐SuppMat.pdf. [file ADVS-13-e23118-s002.pdf]

Supplementary Information

Innovative Metasurface Colorimetric Cell-on-a-Chip Sensor for Continuous, Label-free, Non-Destructive Assessment of Intestinal Barrier Dynamics

Youqian Chen, Wen Li, Jiaying Feng, Yue Shu, Yihui Yang, Yuyan Wang, Rui Li, Hanlin Zhou, Xinfan Yang, Yuxue Guo, Mingqian Chen, Wenjun Hu, Gang Logan Liu, Liping Huang, Yanan Li**

Y. Chen, J. Feng, Y. Shu, Y. Wang, X. Yang, Y. Guo, L. Huang, Y. Li

School of Food Science and Pharmaceutical Engineering, State Key Laboratory of Microbial Technology, Nanjing Normal University, Nanjing 210023, P. R. China.

Y. Chen, W. Li, Y. Yang, M. Chen, W. Hu, G. L. Liu, L. Huang

College of Life Science and Technology, Huazhong University of Science and Technology, 1037 Luoyu Road, Wuhan 430074, P. R. China.

R. Li

School of Biomedical Engineering, Tsinghua University, Beijing 100084, China

H. Zhou

College of life science and technology, Mudanjiang Normal University, Mudanjiang 157011, China.

* Corresponding author.

E-mails:lphuang@nnu.edu.cn (L. Huang), liyanan@njnu.edu.cn (Y. Li)

1 Supplementary Information for the Results and Discussion

1.1 Sensitivity Evaluation of Plasmonic Chips to Refractive Index Changes

Plasmonic nanostructures can also be used for RI sensing. The presence of chemical or biological molecules on these nanostructures alters the dielectric environment surrounding their optical hotspots, thereby affecting the corresponding optical responses used for molecular detection[1]. To assess the sensitivity of the chips to RI changes, we evaluated their responses to varying glycerol concentrations. Both chip types exhibited redshifts in their resonance peaks as the RI increased (**Fig. S1a and d**). After normalization, the spectral shifts were quantified by analyzing optical density variations (OD_{600}–OD_{575}). The PMSPR chip demonstrated greater sensitivity as it detected smaller RI changes (**Fig. S1b and e**). Conversely, the CMSPR chip exhibited a larger response magnitude, suggesting its potential for enhanced signal detection in specific applications (**Fig. S1c and f**).

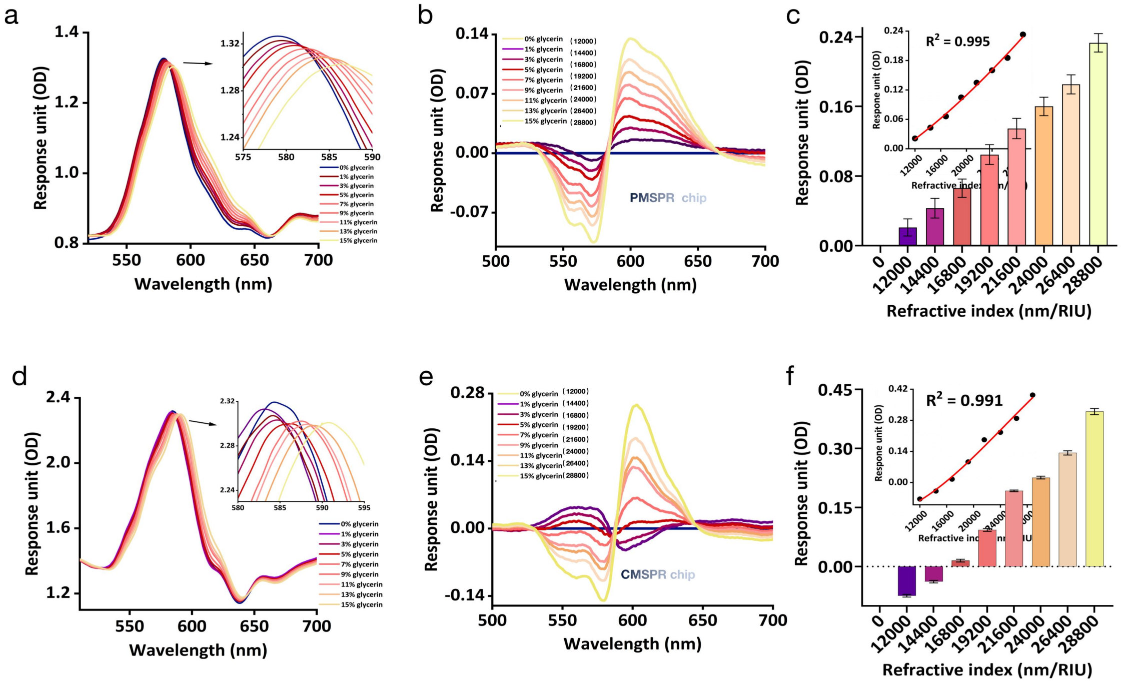

Supplementary Fig. S1 (a) Surface plasmon resonance (SPR) spectra of the gold-only chip, demonstrating redshifts in response to varying refractive indices. (b) Normalized spectral shifts of the gold-only chip. (c) Quantification of peak-to-valley changes, which reveals a linear correlation between changes in refractive index and variations in optical density (OD), with an R^2 value of 0.995. (d) SPR spectra of the gold-silver chip, showing redshifts in response to varying refractive indices. (e) Normalized spectral shifts of the gold-silver chip. (f) Sensitivity characterization of the pure gold MetaSPR chip (PMSPR, 5 nm Ti+60 nm Au) chip.

1.2 Transmittance and Sensitivity Analysis of Plasmonic Chips

This suggests that the pure gold chip has a superior transmittance and was more suitable for the imaging platform. To quantify the changes in the chip transmittance, the transmission spectra of both chips were measured using a range of sucrose concentrations (0.04%–10%) to characterize their responses to RI changes (1.33306–1.34763). The PMSPR chip exhibited a higher original transmittance, whereas the CMSPR chip demonstrated greater normalized transmittance spectral changes **(Fig. S2a and b)**. However, when comparing transmittance changes at the 625 nm wavelength, where optical variations were most pronounced, the PMSPR chip exhibited higher sensitivity. These results confirm that the pure gold chip, with its high transmittance, is better suited for transmissive imaging platforms.

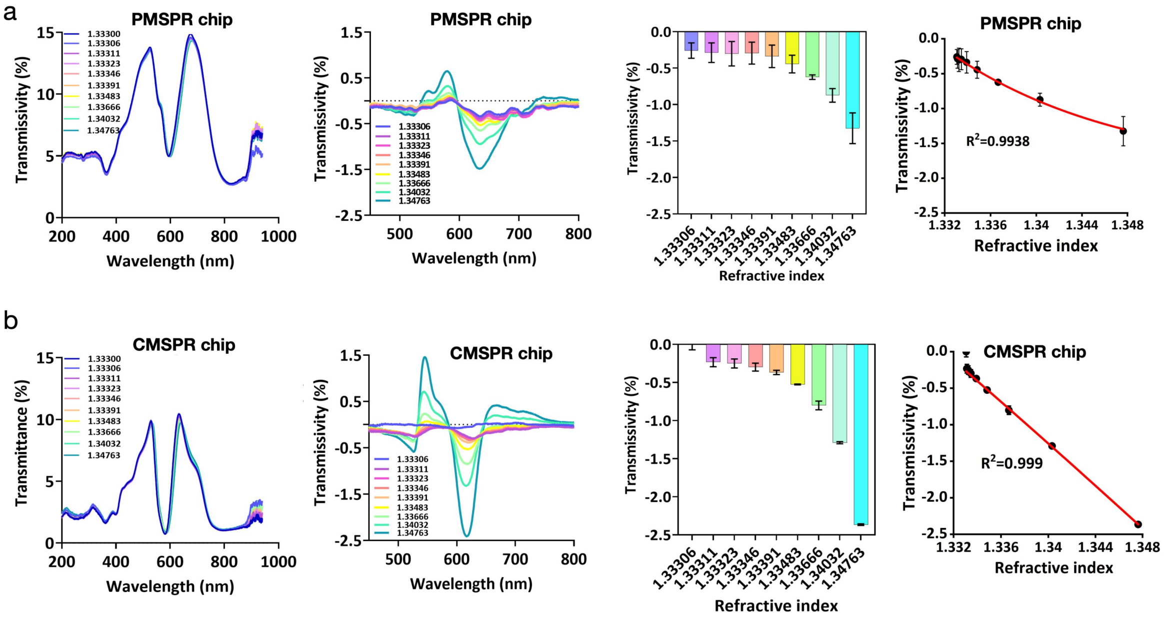

Supplementary Fig. S2 (a-b) Transmittance comparisons for the PMSPR and CMSPR chips in solutions with varying RIs. From left to right: original transmittance spectra, normalized transmittance spectral changes, transmittance comparison at 625 nm, the wavelength exhibiting the maximum change, and standard curve for four-parameter fitting.

1.3 SPR Performance Evaluation through Protein A and IgG Affinity Analysis

We integrated the MetaSPR chip into a microfluidic platform to evaluate its SPR performance through the affinity analysis of Protein A and immunoglobulin G (IgG) (**Fig. S3a**). This experiment utilized the interaction between Protein A and IgG to assess the chip's capability to detect molecular binding events. The affinity results obtained from both the PMSPR and CMSPR chips were consistent with previously reported values in the literature[2], validating the accuracy and reliability of our chip design. Notably, the real-time binding curves of low-concentration samples on the PMSPR chip were clearly distinguishable, indicating its higher sensitivity for detecting low-concentration analytes **(Fig. S3b-3d)**. In contrast, the CMSPR exhibited a larger response unit (RU) value, suggesting a stronger signal output for the same binding event **(Fig. S3e-3g)**. These findings align with the refractive index sensitivity characterization results presented. The higher sensitivity of the PMSPR chip can be attributed to its ability to detect smaller changes in refractive index, while the larger RU value of the CMSPR chip reflects its enhanced signal transduction capability. This complementary performance profile highlights the versatility of MetaSPR chips in various sensing applications.

The PMSPR chip is particularly advantageous for applications that require high sensitivity, such as precise and non-invasive monitoring of cellular behavior. In contrast, the CMSPR chip's larger RU value makes it suitable for applications where a stronger signal is necessary, such as high-throughput screening or multiplexed detection. Overall, this experiment demonstrates the robustness and adaptability of MetaSPR chips for SPR-based sensing. The ability to accurately evaluate the affinity between Protein A and IgG underscores the potential of these chips for studying complex biological interactions, drug-target binding, and other biomedical applications.

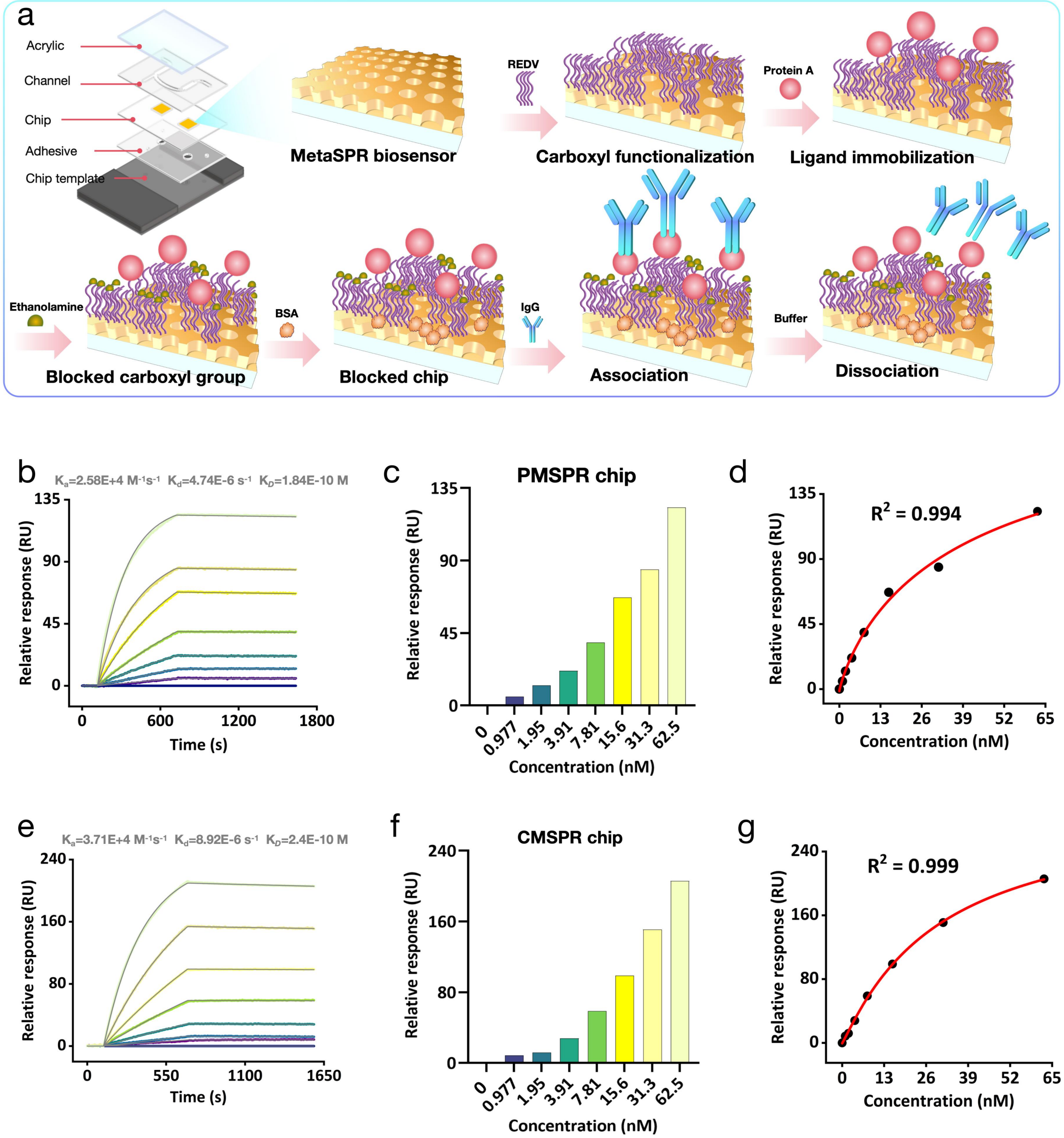

Supplementary Fig. S3 Affinity evaluation of MetaSPR chips through Protein A and IgG interaction. (a) Schematic illustration of the affinity evaluation process. (b) Real-time kinetic curves and fitted results for the PMSPR chip, demonstrating a dissociation constant (K_D) of 1.84E-10 M. (c) A positive correlation between IgG concentration and SPR response signals for the PMSPR chip. (d) Linear relationship between concentration and response for the PMSPR chip, with $R^2 = 0.994$. (e) Real-time kinetic curves and fitted results for the CMSPR chip, indicating a K_D of 2.4E-10 M. (f) A positive correlation between IgG concentration and SPR response signals for the CMSPR chip. (g) Linear relationship between concentration and response for the CMSPR chip, with $R^2 = 0.999$.

1.4 Finite Element Simulation of PMSPR Chips

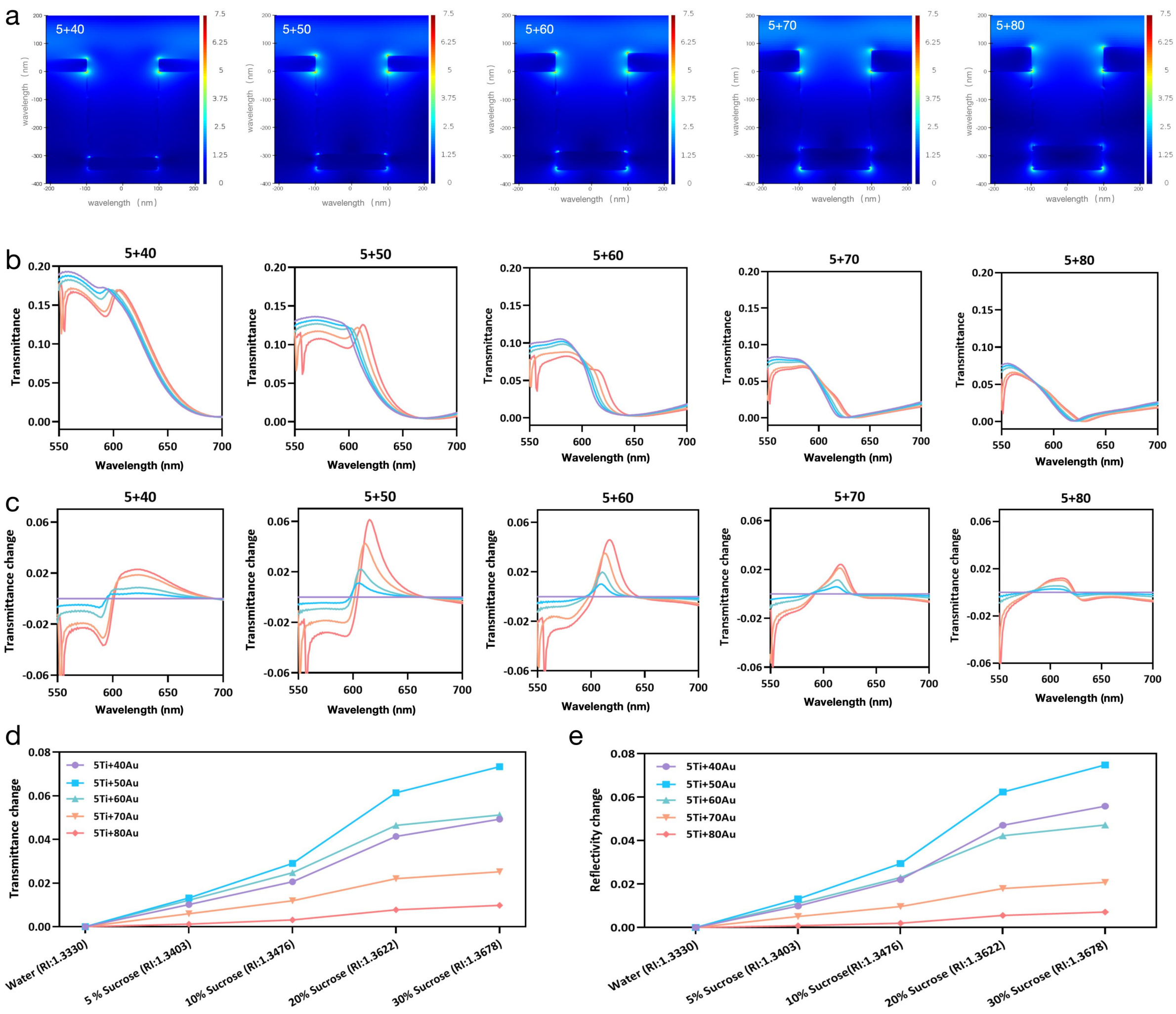

Supplementary Fig. S4 Results of simulating the optimal thickness of the chip using 3D-FDTD software. (a) Simulation of the free electric field active in the pure gold chip with different thicknesses. (b) Simulation of the transmittance of the pure gold chip with different thicknesses as the RI varies. (c) Transmission spectra of chips with different thicknesses are normalized. (d) Comparison of SPR signal magnitude (transmittance $_{peaks}$-transmittance $_{troughs}$) for different thicknesses. (e) Comparison of reflectance effects (reflectance $_{peaks}$ - reflectance $_{troughs}$) for different thicknesses

1.5 Development of Cell-Mimetic Chips and Establishment of Cell Life Cycle Monitoring Models Based on the MetaSPRCS Platform

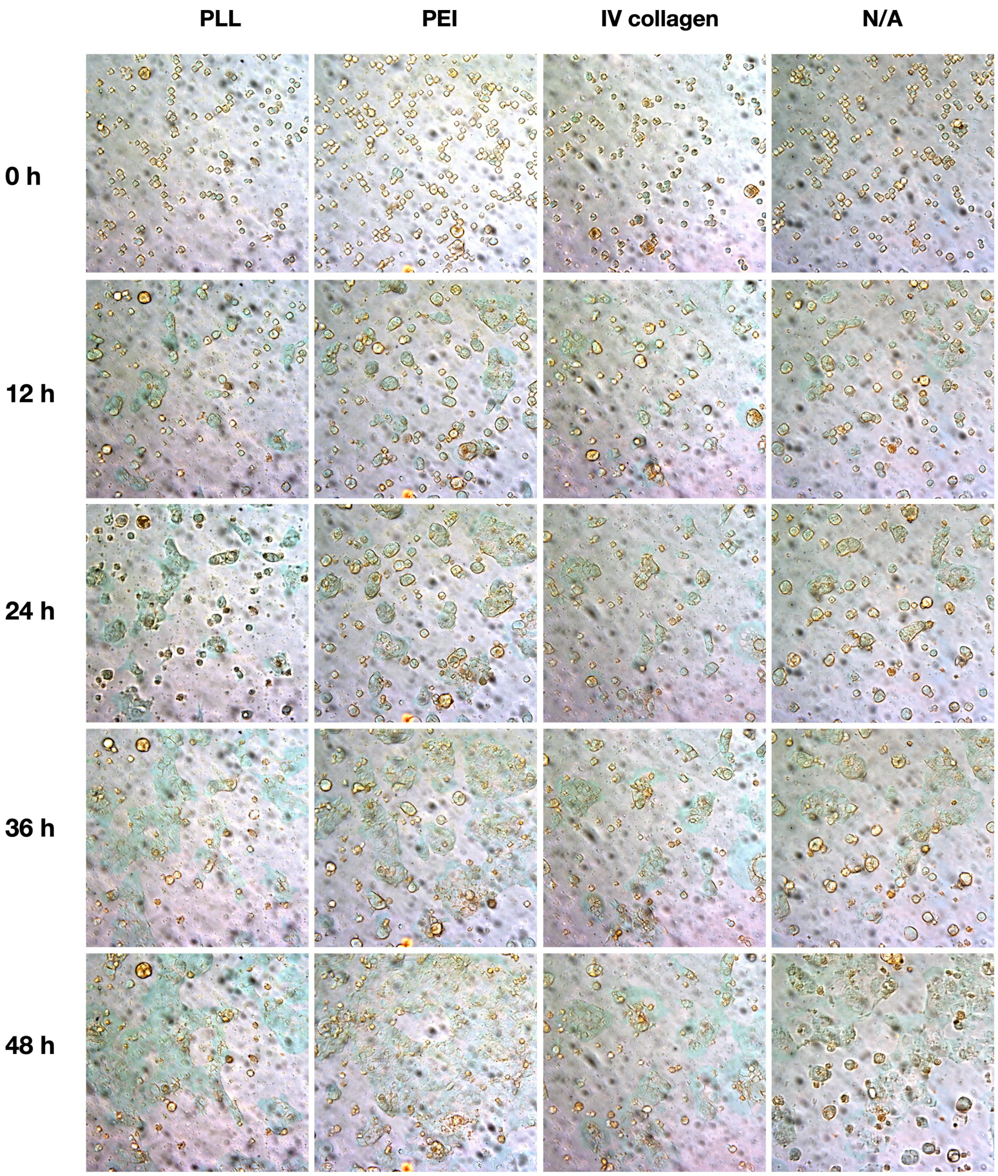

Supplementary Fig. S5 Raw images of cell growth status at different time periods were monitored using microarrays treated with different conditions.

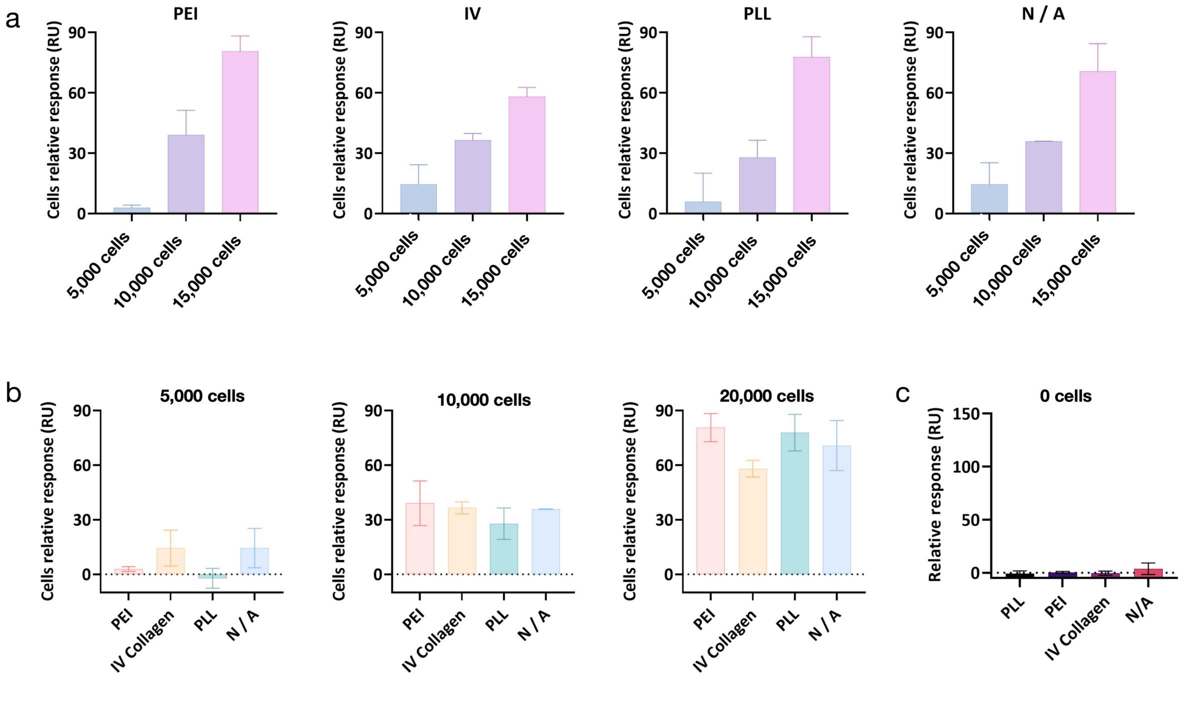

Supplementary Fig. S6 Construction of the bionic chip platform (a) Screening of different modification conditions. (b) Screening of different cell implantation numbers. (c) Non-specific comparison after chemical modification of the chip.

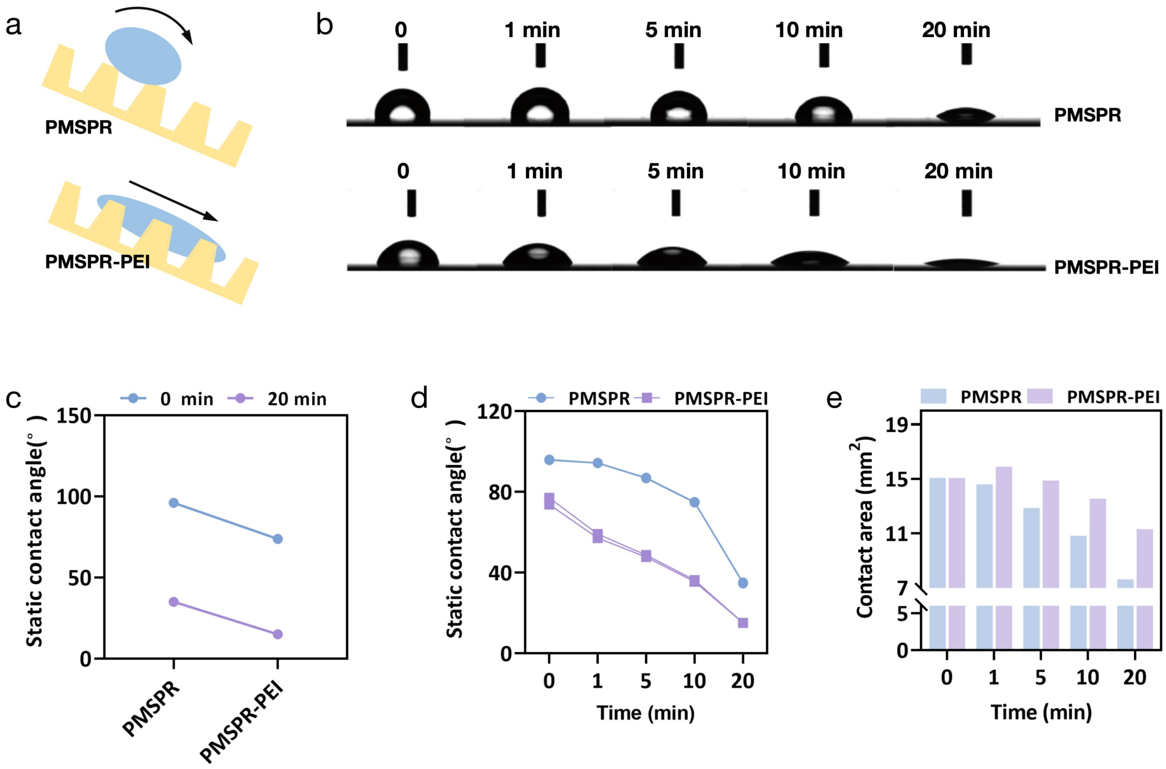

Supplementary Fig. S7 The effect of surface modification and PEI immobilization on the wettability properties of biointerfaces. (a) Schematic of the sliding angle of a 5 μL water droplet on unmodified PMSPR chip and PMSPR-PEI. (b) Observation of the 5 μL water droplet's evaporation on unmodified PMSPR and PMSPR-PEI chip, to assess the transition from the Cassie-Baxter state to the Wenzel state of wettability. The water droplet on the PMSPR chip maintained a contact angle (CA) greater than 90° for up to 5 min due to the excessive air gaps created by the substrate's hierarchical texture. The CA dropped only in 20 min, leading to complete evaporation of the droplet. In contrast, the water droplet on the PMSPR-PEI substrate was immediately absorbed upon dispensing, indicating a lack of transition through the Cassie-Baxter state. (c) Comparing the sliding angle of 5 μL water droplet onto unmodified PMSPR chip, as well as PMSPR-PEI. (d) Measuring the CA of 5 μL water on PMSPR chip and PMSPR-PEI chip (n = 2, independent replicates). (e) Measure the diffusion area of 5 μL of water on the PMSPR chip and the PMSPR-PEI chip.

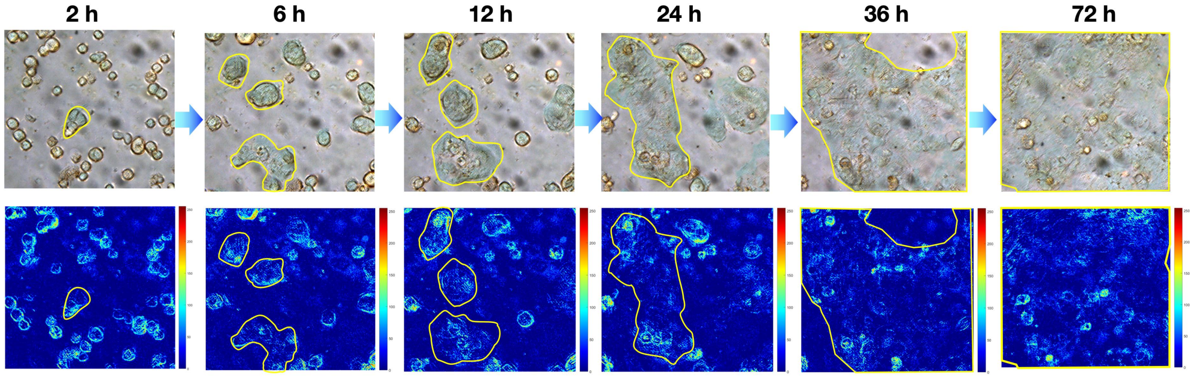

Supplementary Fig. S8 Imaging changes during barrier layer formation of PEI-modified bionic chips. Above is the original image and below is the extracted SPR signal.

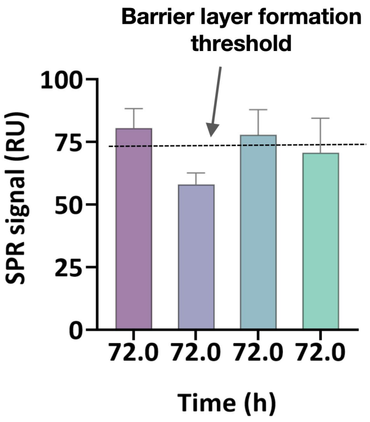

Supplementary Fig. S9 SPR signal threshold for barrier layer formation based on the biochip. The experiments were repeated four times using different batches of chips, with three replicates per batch. The threshold was calculated as the average of all the signals.

1.6 Evaluation of Alcohol-Induced Intestinal Barrier Damage Based on MetaSPRCS Modeling

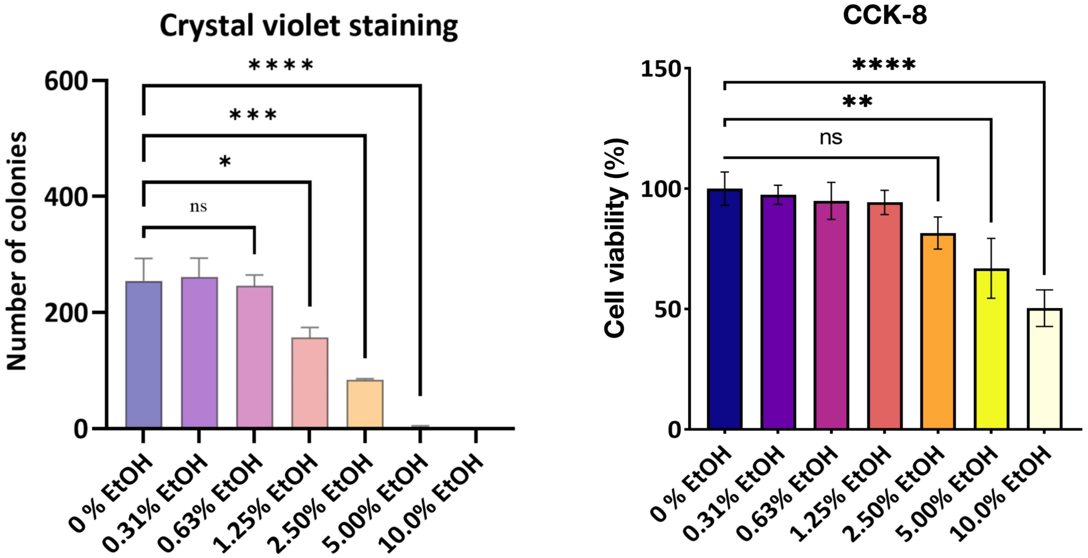

Supplementary Fig. S10 Traditional endpoint method to assess the damaging effects of alcohol on the cellular barrier.

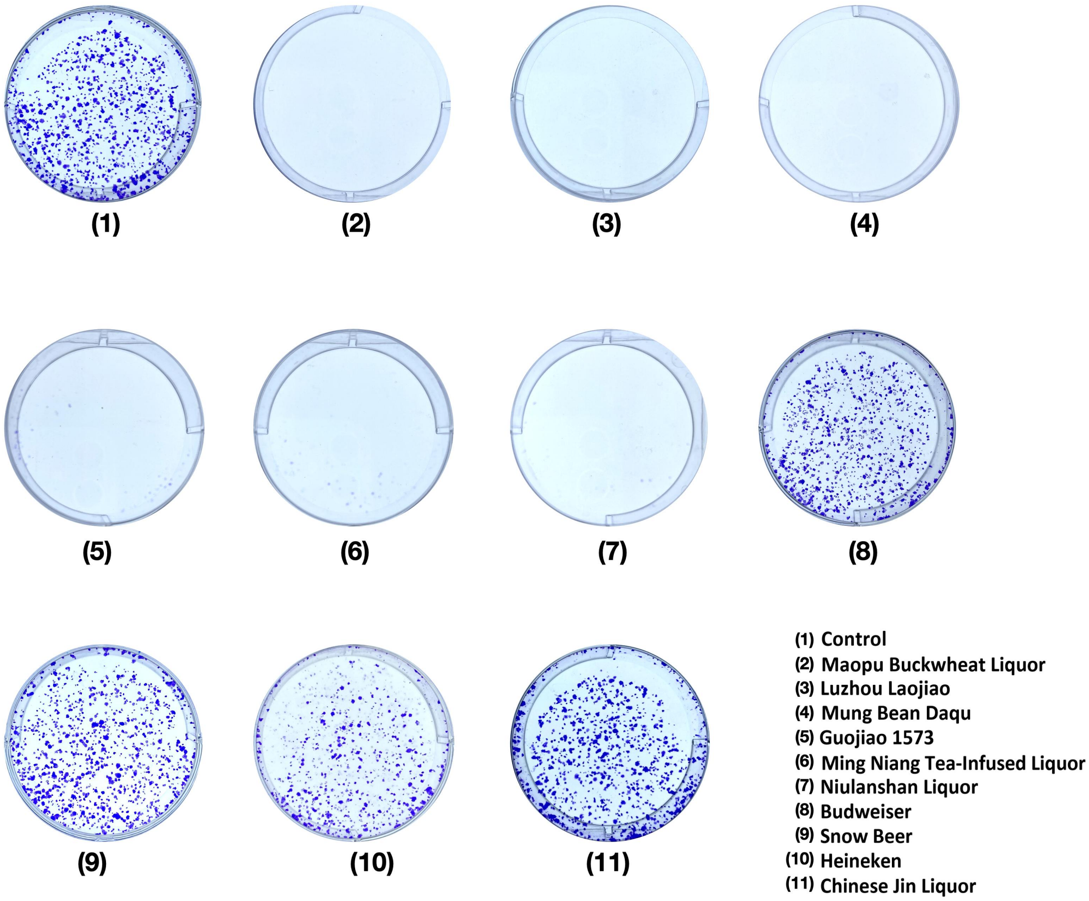

Supplementary Fig. S11 Evaluation of cellular barrier damage by different wines using crystal violet staining assay.

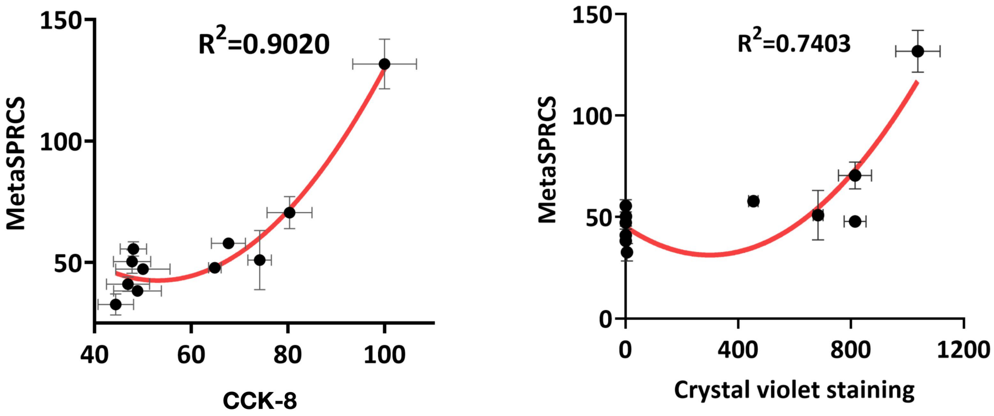

Supplementary Fig. S12 Correlation of the results of the MetaSPRCS platform versus traditional methods for evaluating damage to the cellular barrier by different wines separately.

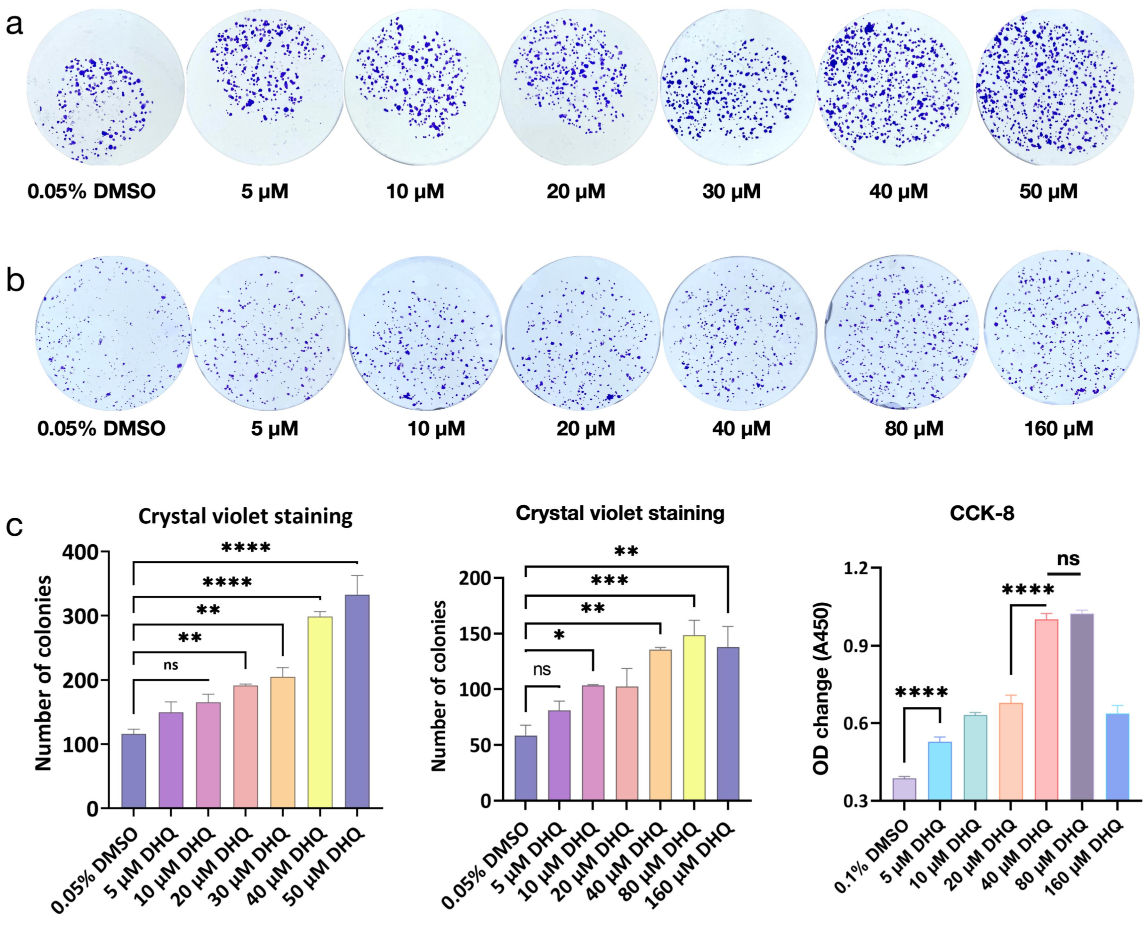

Supplementary Fig. S13 Evaluation of the effect of different concentrations of DHQ repair. (a) Effect of low concentration of repair, crystal violet staining. (b) The effect of high concentration repair with crystal violet staining. (c) Comparative cellular integrity assessment by crystal violet staining vs. CCK-8 live-cell monitoring.

1.7 Animal Experimentation and Establishment of *In Vivo-In Vitro* Correlation

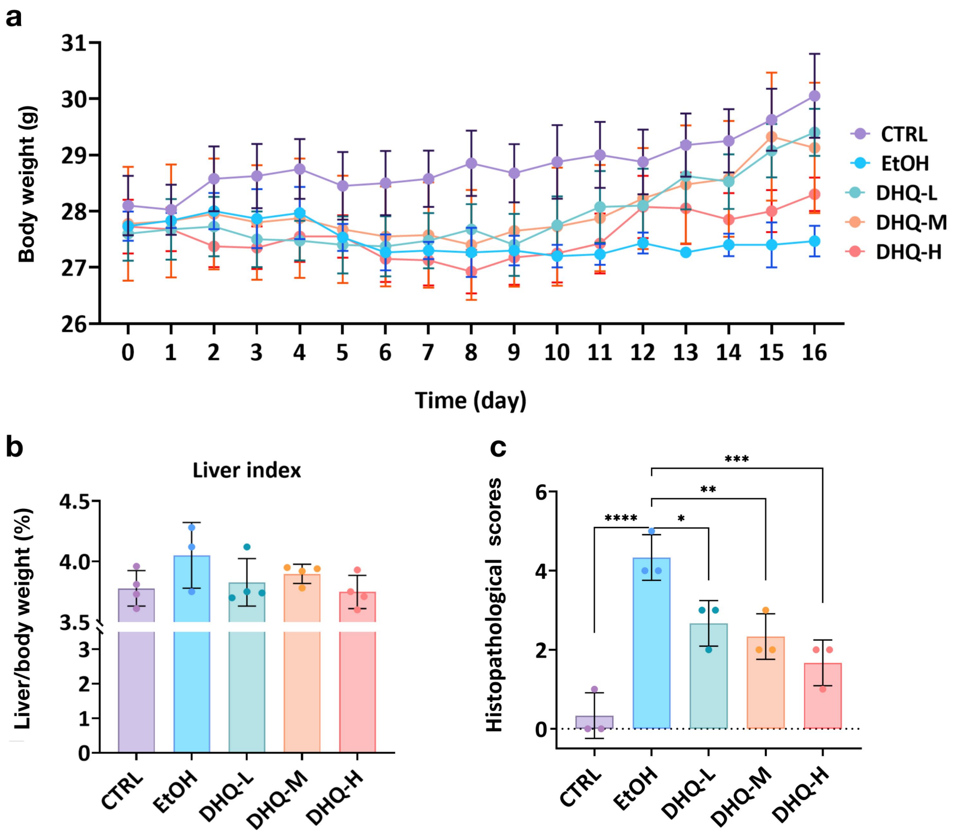

Supplementary Fig. S14 (a) Growth curves and final body weight of the mice in the five treatment groups during experiment periods. (b) Liver weight among different experimental groups. (c) Histopathological scores in the mice of five treatment groups.

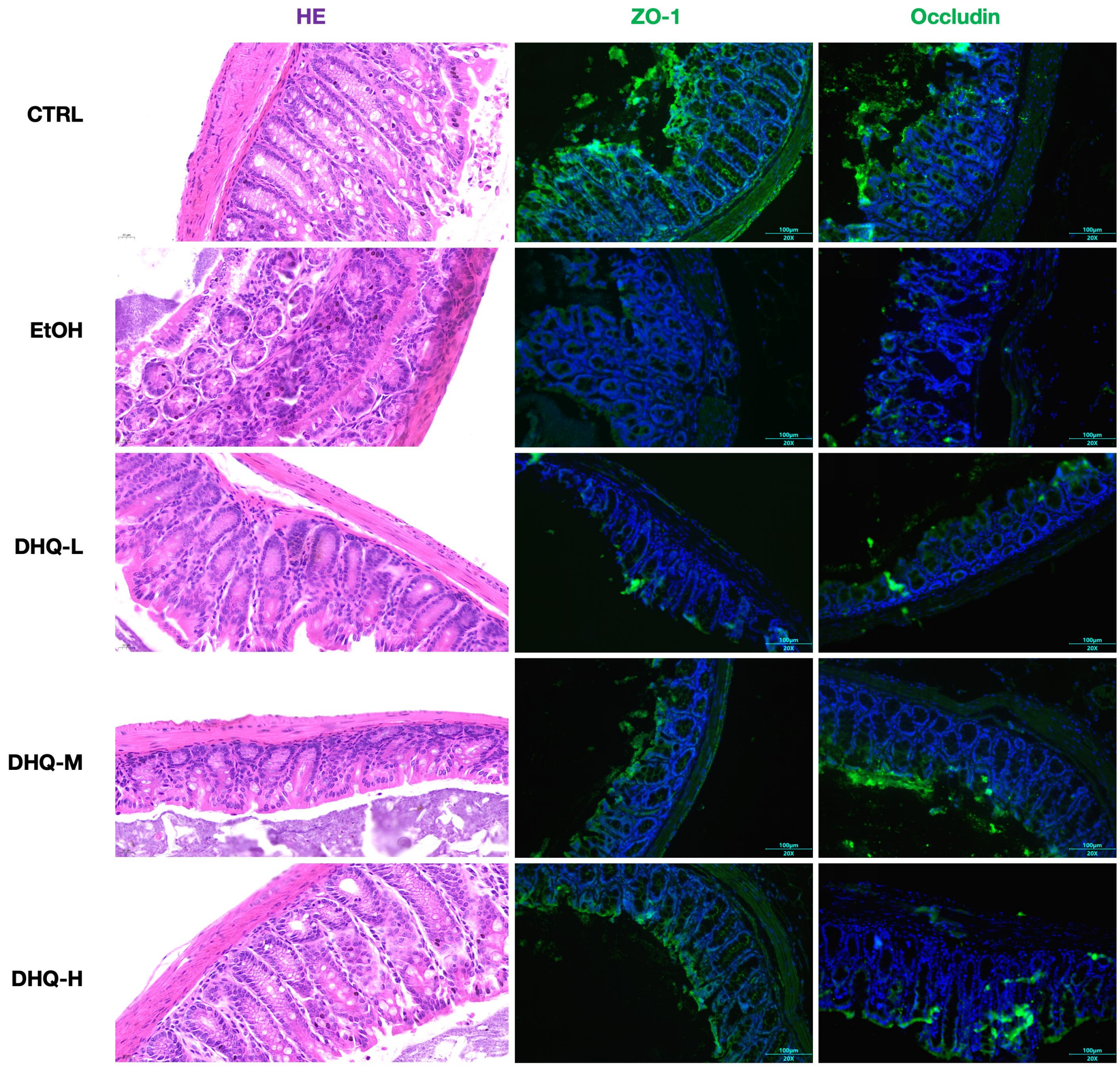

Supplementary Fig. S15 Representative image of H&E staining and immunofluorescence image of ZO-1 and Occludin in colon tissue of the four treatment groups.

2 Supplementary Information for the Experimental Section

2.1 Materials

Caco-2 cells (RRID: CVCL_0025) were obtained from Wecare Probiotics Co., Ltd. (Suzhou, China). Cell culture plates and cryovials were purchased from Corning Inc. (Corning, NY, USA). Dulbecco's Modified Eagle Medium (DMEM) (with antibiotics) and Trypsin − EDTA solution (0.25%) were purchased from KeyGEN BioTECH (Jiangsu, China). Fetal bovine serum (FBS) was purchased from Royacel (Gansu, China). Sterile DMSO (A3672.0050) was purchased from PanReac AppliChem (Beijing, China). Protein A was purchased from Sino Biological Inc. (Beijing, China). The mouse anti-human Fc IgG, bovine serum albumin (BSA), and Taxifolin (Dihydroquercetin, DHQ) were purchased from Beijing Solarbio Co. Ltd. (Beijing, China). Tween 20 was purchased from Sigma-Aldrich (St. Louis, MO, USA). Phosphate-buffered saline (PBS) was purchased from Thermo Fisher Scientific Inc. (Waltham, MA, USA). No further purification was performed on the chemicals used. The WeSPR™ Cell Analyzer, WeSPR™ 100X and WeSPR One Auto™ instruments were purchased from Xlement (Wuhan, China). Budweiser Beer was purchased from Budweiser Beer Co., Ltd. (Wuhan, China). Heineken Beer was purchased from Snow Beer Co., Ltd. (Guangzhou, China). Snow Beer was purchased from China Resources Snow Beer Co., Ltd. (Liaoning, China). Jin liquor was purchased from Jin liquor Co., Ltd. (Hubei, China). Maopu liquor was purchased from Maopu liquor Co., Ltd. (Hubei, China). Niulanshan liquor was purchased from Beijing Shunxin Agricultural Co., Ltd. (Beijing, China). Luzhou Laojiao was purchased from Luzhou Laojiao Co., Ltd (Sichuan, China).

2.2 Comprehensive Performance Evaluation of MetaSPR Biosensor Chips

Comparative evaluation of biosensor chip responsiveness via optical density response to refractive index variations. This study aimed to characterize the effect of refractive index variations on the SPR response signals. Glycerol-water solutions with varying concentrations (0%, 1%, 3%, 5%, 7%, 9%, and 11% w/v) were prepared to assess the sensitivity of both sensor chips to changes in surface substances. Subsequently, 50 μL aliquots of each glycerol solution were introduced into individual wells of the biosensor chip. Absorption spectra were measured across the 500–700 nm wavelength range using a microplate reader operating in full-wavelength scanning mode.

Evaluation of chip performance by transmitted light response to RI changes. The sensor chip was mounted onto a chip carrier for optical characterization. Sucrose solutions were prepared in a concentration gradient (0%, 0.039%, 0.078%, 0.15625%, 0.3125%, 0.625%, 1.25%, 2.5%, 5%, 10% w/v) and sequentially tested, starting with the 0% solution. Transmittance measurements were conducted using a transmission spectrometer to evaluate the intensity of transmitted light through the functionalized chip.

Variations in intensity at 625 nm, corresponding to the wavelength of maximum optical response variation, were quantified and presented as bar graphs. A comparative analysis of light intensity shifts across the concentration gradients was performed to assess chip sensitivity.

Sensitivity assessment of protein-protein interactions. Sensitivity evaluation of protein-protein interactions was conducted utilizing a series of methodical steps. Initially, a 50 μL volume of thioglycolic acid at a concentration of 5 μM was applied to the chip surface and allowed to incubate for 16 h at 4 °C, facilitating the formation of a carboxylated surface. Following this, the carboxyl groups on the chip were activated through the application of a 1:1 mixture of 50 mg/mL N-hydroxysuccinimide (NHS) and 50 mg/mL 1-ethyl-3-(3-dimethylaminopropyl) carbodiimide (EDC), with the activation process lasting for 30 min. The chip was subsequently rinsed twice with ddH2O. A 2 μL aliquot of a peptide solution (REDV) at a concentration of 10 μg/mL was then deposited at the center of the chip well and incubated overnight at 4 °C to construct the carboxylated peptide sensor. The peptide carboxylated sensors were subsequently positioned in the card slot and hydrated with phosphate-buffered saline with Tween (PBST, pH 7.4) at a flow rate of 10 μL/min to ensure the stabilization of surface properties. This was succeeded by a series of surface functionalization steps, which included EDC/NHS activation (10 μL/min), immobilization of the ligand protein (50 μg/mL Protein A in MES, 10 μL/min), and the application of a blocking solution (50 mM ethanolamine, 1% BSA, 20 μL/min) to reduce nonspecific binding interactions. For the profiling of analyte interactions, IgG solutions at varying concentrations of 0, 0.156, 0.313, 0.625, 1.25, 2.5, 5, and 10 μg/mL were injected through multiple channels at a rate of 40 μL/min. The real-time binding dynamics were monitored using the WeSPR One Auto™ system (version 5.3.0, Xlement). The optical intensity modulations at 625 nm, which corresponded to peak sensitivity responses, were quantified to evaluate concentration-dependent signal variations.

2.3 Operation Methods for Comparative Experiments

CCK-8. To evaluate the cytotoxic effects of ethanol at varying concentrations on cells, Caco-2 cells were seeded in 96-well cell culture plates (15,000 cells/well in 100 μL DMEM medium). Following a 12 h incubation period for cell attachment and proliferation, the cells were treated with ethanol at concentrations of 10%, 5%, 2.5%, 1.25%, 0.63%, and 0.31% for 12 h. Subsequently, 10 μL of CCK-8 reagent was added to each well, and the plates were incubated at 37 °C under 5% CO_2 for 2 h. Absorbance at 450 nm was measured using a microplate reader. To assess the cytotoxic effects of various alcoholic beverages on cells, cells were allowed to adhere and proliferate using the aforementioned method. Subsequently, different types of alcoholic beverages (Mao Pu, Luzhou Laojiao, Lüdou Daqu, Guojiao1573, Ming Niang, Niulanshan, Budweiser, Snow, Heineken, Jingjiu) were mixed with DMEM culture medium at a 1:1 ratio and added to the 96-well plate, followed by incubation for 12 h. The CCK-8 reagent was then introduced according to the previously described protocol, and absorbance values at 450 nm were measured to

quantify cellular viability. To evaluate the reparative effects of DHQ on ethanol-induced intestinal barrier injury, cells were first allowed to adhere and proliferate using the aforementioned protocol. Subsequently, the wells were challenged with 2.5% ethanol for 12 h to induce cellular damage. Following ethanol treatment, the medium was aspirated, and DHQ was supplemented at concentrations of 50 μM, 40 μM, 30 μM, 20 μM, 10 μM, and 5 μM. After a 24 h recovery period, the CCK-8 reagent was added according to the established method, and absorbance at 450 nm was measured to assess cellular viability.

Crystal violet staining. To evaluate the cytotoxic effects of ethanol at varying concentrations on cells, Caco-2 cells were seeded in 6-well plates at 2,000 cells/well with 2 mL culture medium per well. After colony formation, the cells were treated with ethanol at concentrations of 10%, 5%, 2.5%, 1.25%, 0.63%, and 0.31% for 12 h. Following treatment, the supernatant was removed and cells were washed three times with PBS. Fixed with 1.5 mL of 4% paraformaldehyde for 15 min, then rinsed three times with PBS. Cells were stained with crystal violet solution for 15 min, followed by thorough washing with deionized water to remove residual dye. Air-dried samples were documented by imaging. To assess the cytotoxic effects of various alcoholic beverages on cells, cells were seeded following the established protocol. Post-clonogenic growth, different types of alcoholic beverages (Mao Pu, Luzhou Laojiao, Lüdou Daqu, Guojiao1573, Ming Niang, Niulanshan, Budweiser, Snow, Heineken, Jingjiu) were mixed with DMEM medium at a 1:1 ratio and introduced into the wells. After 12 h of incubation, cells were fixed and stained according to the aforementioned methodology for quantitative analysis of colony survival. When evaluating the reparative effects of DHQ on intestinal cell barrier damage, following clonogenic formation, cells were challenged with 2.5% ethanol for 12 h to induce cellular damage. After ethanol exposure, the medium was aspirated, and fresh medium supplemented with DHQ at concentrations of 50, 40, 30, 20, 10, and 5 μM was administered. Following a 24 h recovery period, cells were fixed and stained according to the aforementioned methodology to assess morphological restoration.

References

[1] H. S. Yun, X. Liu, H. Salihoglu, Z. Li, S. Shen, *Materials Today Physics* **2024**, *42*, 101369, https://doi.org/https://doi.org/10.1016/j.mtphys.2024.101369.

[2] Y. Chen, H. Fan, R. Li, H. Zhang, R. Zhou, G. L. Liu, C. Sun, L. Huang, *Advanced Science* **2024**, *11* (46), 2404559, https://doi.org/https://doi.org/10.1002/advs.202404559.
